# Comparative Risk of Renal Adverse Events in Patients Receiving Immune Checkpoint Inhibitors: A Bayesian Network Meta-Analysis

**DOI:** 10.3389/fonc.2021.662731

**Published:** 2021-06-16

**Authors:** Kang Liu, Zhongke Qin, Xueqiang Xu, Ting Li, Yifei Ge, Huijuan Mao, Changying Xing

**Affiliations:** Department of Nephrology, Jiangsu Province Hospital (The First Affiliated Hospital of Nanjing Medical University), Nanjing, China

**Keywords:** treatment regimen, cancer, acute kidney injury, renal adverse events, immune checkpoint inhibitors

## Abstract

**Background:**

Immune checkpoint inhibitors (ICIs) have brought a paradigm shift to cancer treatment. However, little is known about the risk of renal adverse events (RAEs) of ICI-based regimens, especially ICI combination therapy.

**Methods:**

We carried out a network meta-analysis of randomized controlled trials (RCTs) to compare the risk of RAEs between ICI-based regimens and traditional cancer therapy, including chemotherapy and targeted therapy. Subgroup analysis was conducted based on tumor types.

**Results:**

Ninety-five eligible RCTs involving 40,552 participants were included. The overall incidence of RAEs, grade 3–5 RAEs, acute kidney injury (AKI), and grade 3–5 AKI was 4.3%, 1.2%, 1.3%, and 0.8%, respectively. Both ICI-based treatment regimens and traditional cancer therapy showed significantly higher risk of RAEs and AKI than the placebo. Among ICI monotherapy, anti-PD-1 (RR: 0.51, 95%CI: 0.29–0.91) was significantly safer than anti-CTLA-4 in terms of RAEs. Anti-CTLA-4 showed significantly higher toxicity than anti-PD-1 (RR: 0.33, 95%CI: 0.14-0.77), anti-PD-L1 (RR: 0.38, 95%CI:0.16-0.91), and anti-PD-1 plus anti-CTLA-4 (RR: 0.32, 95%CI: 0.12-0.87) in terms of grade 3-5 RAEs. The difference was not significant between ICI monotherapy and traditional cancer therapy, except that targeted therapy seemed the least toxic therapy in terms of the incidence of AKI. Anti-CTLA-4 plus anti-PD-1 were associated with higher risk of RAEs than anti-PD-1 (RR: 1.61, 95%CI: 1.02–2.56). The difference was not significant between other dual ICI regimens and ICI monotherapy in terms of RAEs and AKI. ICI plus chemotherapy showed increased risk of both RAEs and AKI compared with ICI monotherapy, chemotherapy, and targeted therapy. The overall results remained robust in the meta-regression and sensitivity analyses.

**Conclusions:**

Among ICI monotherapy, anti-CTLA-4 appeared to be associated with increased toxicity, especially in terms of grade 3–5 RAEs. Anti-CTLA-4 plus anti-PD-1 were associated with higher risk of RAEs than anti-PD-1. However, the difference was not significant between other dual ICI regimens and ICI monotherapy in terms of RAEs and AKI. ICIs plus chemotherapy seemed to be the most toxic treatment regimen in terms of RAEs, AKI, and grade 3–5 AKI.

**Systematic Review Registration:**

PROSPERO, identifier CRD42020197039.

## Introduction

Immune checkpoint inhibitor (ICI) therapy has unveiled a new era in cancer treatment, yielding an unprecedented and robust response in the treatment of different malignancies. These ICIs release inactive immune responses by blocking specific down-regulators of the immune response including cytotoxic T-lymphocyte antigen 4 (CTLA-4) and programmed cell death 1 (PD-1) and its ligand, programmed cell death ligand 1 (PD-L1) (1). Although these regulators mediate an inhibitory effect on T cell response, they exert their biological effect *via* different mechanisms and on different sites (2). CTLA-4, expressed on the surface of T cells, slows down the CD4+ and CD8+ cells’ activation by inhibiting the co-stimulatory signaling pathway within lymphoid organs (3, 4). PD-1, a protein receptor expressed by T cells, B cells, NK cells, and several other tumor-infiltrating lymphocytes, acts within peripheral tissues (5, 6). It functions by binding to its ligand PD–L1 on the antigen-presenting cells, leading to T cells exhaustion and inhibiting their capacity of activation and differentiation (7, 8). Therefore, by targeting these immune checkpoints, ICIs can reinvigorate T cell activity and augment antitumor immunity.

Since 2011, seven immune checkpoint-directed antibodies have been approved by the US Food and Drug Administration (FDA), and both ICI monotherapy and combination therapy have achieved great success in a variety of cancers (9–11). To improve patients’ response, an increasing number of studies are focusing on regimens combining ICIs with traditional cancer therapies such as chemotherapy and targeted therapy. Based on Keynote-189, ICIs in combination with chemotherapy is now considered the standard–of–care for metastatic non-small cell lung cancer (NSCLC) (12). Most recently, ICIs combined with tyrosine kinase inhibitors has been approved for the treatment of renal cell carcinoma and endometrial cancer (13, 14).

The successful antitumor effects of ICIs are limited by the unique side effects termed immune-related adverse events (irAEs). Similar to autoimmune diseases, irAEs can affect multiple organ systems in the body. Dermatological complications are the most common, followed by gastrointestinal distress, hepatotoxicity, and endocrinopathies (15, 16). Renal toxicity is less common; however, it is attracting increasing attention as the use of ICIs continues to expand. The incidence of ICI–associated acute kidney injury (AKI) is estimated to range from 1.4% to 4.9%, with dual ICI regimens carrying an increased risk when compared with monotherapy with anti-CTLA-4, anti-PD-1, or anti-PD-L1 (17–20). Although a broad spectrum of renal lesions have been reported, tubulointerstitial nephritis (TIN) is recognized as the most common renal pathology (18, 20, 21).

The incidence and risks of renal adverse events (RAEs) in ICI monotherapy and dual ICI regimens are relatively well recognized; however, there is a new urgent need to understand the incidence and risks of ICIs in combination with traditional cancer therapy, including chemotherapy and targeted therapy. Thus, we conducted this network meta-analysis to explore the risk of RAEs in patients with ICI monotherapy and combination therapy.

## Methods

This network meta-analysis was conducted according to a prespecified protocol and followed the Preferred Reporting Items for Systematic Reviews and Meta-Analyses (PRISMA) guidelines (**Supplementary Table 1**) (22). Ethics committee approval was not required for this study design. The study was registered with PROSPERO (number: CRD42020197039).

### Data Sources and Searches

A systematic search of the literature was conducted in PubMed, Embase, and the Cochrane Library (before June 1, 2020) without imposing any language restrictions. The search strategy is detailed in **Supplementary Table 2**. As the publication bias caused by unpublished data can significantly interfere with the relative efficacy of the network meta-analysis and modify the rankings, we also searched the ClinicalTrials.gov website (https://clinicaltrials.gov/) for unpublished or ongoing trials. Furthermore, we manually searched the reference lists of retrieved records and clinical trial registries to identify additional studies.

### Study Selection

Studies were included if they met all of the following criteria: (a) randomized clinical trials (RCTs) of patients with cancer; (b) at least one treatment group received an FDA-approved ICI, as monotherapy or combined with another ICI or traditional cancer therapy; and (c) reported data of RAEs in each group. When multiple publications covering the same study were identified, we included the one with the most recent and comprehensive data. Studies that failed to meet the above criteria were excluded. We also excluded reviews, meetings, conference abstracts, and case reports.

Two investigators (ZQ and KL) independently evaluated the title and abstract of retrieved reports, screened their full text for eligibility, and further assessed risk of bias. Clinical trials with results from ClinicalTrials.gov were also identified and included. Any discrepancy during the processes was resolved by discussion with a third reviewer (XX).

### Data Extraction and Quality Assessment

ZQ entered data into an electronic spreadsheet (Microsoft Excel). KL independently checked the data and resolved disagreements by discussion. The primary outcome of the review was RAEs, which were defined as adverse events reported in the form of increased blood creatinine, decreased renal creatinine clearance, decreased urine output, oliguria, anuria, glomerulonephritis, TIN, nephritis, autoimmune nephritis, renal tubular acidosis, nephropathy toxic, nephrotic syndrome, glomerulosclerosis, kidney fibrosis, renal failure, acute renal failure, prerenal failure, postrenal failure, renal injury, renal impairment, and chronic kidney disease (CKD). Other outcomes were classified as grade 3–5 RAEs, AKI, and grade 3–5 AKI. AKI was defined according to the Kidney Disease Improving Global Outcomes (KDIGO) sCr criteria and the Common Terminology Criteria for Adverse Events (CTCAE), specifically as a >0.3 mg/dL increase or a >1.5-fold rise in serum creatinine from baseline. We defined the grading of adverse events on the basis of the Common Terminology Criteria for Adverse Events (CTCAE) applied in individual clinical trials. When different doses of the same ICI regimen were used in a trial, we chose the one in line with the approval dose of the FDA (**Supplementary Table 3**). We did not distinguish between different chemotherapeutic or targeted drugs and considered them as one group in a trial. Quality was assessed independently by researchers in a blinded fashion. We assessed the sources of bias using the Cochrane Collaboration risk-of-bias tool (23).

### Statistical Analysis

Conventional pairwise meta-analysis was initially performed taking into account the available head-to-head comparisons. We used risk ratio (RR) and its 95% credible intervals to estimate the risk of RAEs of different regimens. A standard random-effects model was applied because of the expected variation among various regimens to provide more conservative estimated effects. Statistical heterogeneity was assessed using the I-squared (I^2^) statistic (24). The Bayesian network meta-analysis was conducted using random-effects generalized linear models based on the Markov chain Monte Carlo method (25). Each of the four chains was simultaneously run for 50,000 burn-ins and 100,000 inference iterations per chain to obtain posterior distribution. The convergence of the model was detected using the Gelman–Rubin method combined with a density plot and tract plot (26). For all outcomes, we summarized the evidence by drawing a network relation graph. The RAEs of different treatment regimens were ranked according to surface under the cumulative ranking (SUCRA) curve (27). League tables summarized all possible comparisons in the network, which indicated whether the estimated differences among different regimens were statistically significant. Model fit was assessed by calculating the deviance information criterion (DIC) as the sum of the posterior mean of the residual deviance and leverage pD. The transitivity assumption was evaluated by comparing the distribution of potential effect modifiers (mean age, sex ratio, sample size, and year) across treatment comparisons. In our analysis, global inconsistency was evaluated by the design-by-treatment interaction approach (28). To check the assumption of local consistency, the loop-specific approach and node-splitting method were used (29). We adopted the tau-squared (τ^2^) test to evaluate the extent of heterogeneity for each outcome. Additionally, meta-regression analyses and sensitivity analyses were conducted to explore the sources of heterogeneity and ensure the validity and robustness of the findings. Further, to probe the rankings of all treatment regimens for the secondary outcomes, we conducted subgroup analyses based on different outcome definitions (grade 3–5 RAEs, AKI and grade 3–5 AKI) and cancer types. Publication bias was assessed by examining the potential presence of small-study effects *via* the visual inspection of comparison-adjusted funnel plots (29). Pairwise meta-analysis was conducted using Stata, version 13 (StataCorp LP), and NMA within the Bayesian framework was conducted using R software, version 3.5.3 (R Foundation for Statistical Computing, Vienna, Austria), with the packages “gemtc 0.8-2” recalling JAGS (version 4.3.0) (30). P<0.05 was considered to indicate statistical significance.

## Results

### Study Characteristics

**Figure 1:**
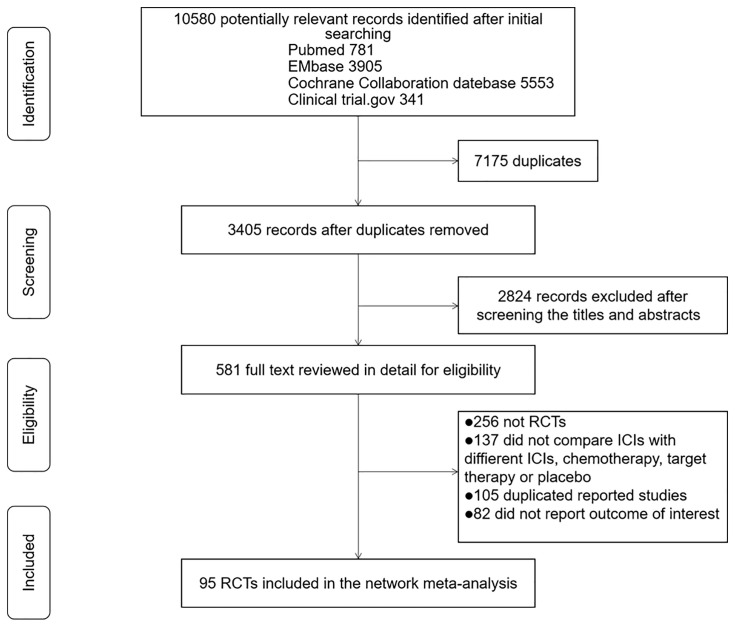
PRISMA flow chart of literature search and selection.

The initial literature search yielded 10,580 records, of which 581 records were retrieved for detailed assessment (**Figure 1**). Finally, a total of 95 eligible RCTs involving 40,552 participants were selected in the network meta-analysis. The essential baseline characteristics of these RCTs are presented in **Supplementary Table 4**. Sixteen included trials assessed ≥3 treatment regimens, which were made by pairwise comparison in the meta-analysis. The mean age for participants ranged from 47.1 to 74 years, and the proportion of male subjects was 66% in the total population. The median number of study participants was 361. The overall incidence of RAEs, grade 3–5 RAEs, AKI, and grade 3–5 AKI was 4.3% (1,756 of 40,552 patients from 95 studies), 1.2% (473 of 40,290 patients from 90 studies), 1.3% (348 of 27,009 patients from 63 studies) and 0.8% (229 of 26,819 patients from 62 studies), respectively. **Supplementary Figure 1** shows the incidence of nephrotoxicity of different kinds of treatment regimens. The anti-PD-1 plus chemotherapy, anti-PD-1 plus anti-CTLA-4, and anti-PD-1 plus targeted therapy were associated with relatively higher rate of RAEs, grade 3–5 RAEs, AKI, and grade 3–5 AKI than other regimens.

### Risk of Bias Assessment and Publication Bias

Overall, the quality of the trials was acceptable, with 96.8% of studies at low risk of bias for the random sequence generation, 80% at low risk of bias for allocation concealment, 95.8% at low risk of bias for incomplete outcome data, and 98.9% at low risk of bias for selective reporting. However, most of the studies were reported to have an unclear risk of bias in blinding participants and personnel (73.7%) and blinding of outcome assessment (63.2%). The risk of bias for each included trial is detailed in **Supplementary Figure 2**. In addition, inspection of comparison-adjusted funnel plots revealed no distinct asymmetry and therefore no significant risk of small-study effects was recognized (**Supplementary Figure 3**).

### Conventional Pairwise Meta-Analysis

The results of the pairwise meta-analysis in terms of RAEs are shown in **Supplementary Table 5**. Anti-PD-1 plus chemotherapy (RR, 3.13; 95% CI, 2.08-4.76), anti-PD-1 plus targeted therapy (RR: 1.75, 95%CI: 1.06-2.94), anti-PD-1 plus anti-CTLA-4 (RR: 2.04, 95%CI: 1.10-3.70) and chemotherapy plus targeted therapy (RR: 2.33, 95%CI: 1.19-4.55) showed remarkably higher toxicity than anti-PD-1. Furthermore, anti-PD-1 plus chemotherapy was associated with significantly increased toxicity when compared with chemotherapy (RR: 1.99, 95%CI: 1.03-3.85) and chemotherapy plus targeted therapy (RR: 1.95, 95%CI: 1.15-3.30). The results of available direct comparisons and testing heterogeneity (I^2^, τ^2^, and Q) of different treatment regimens are listed in **Supplementary Table 5**. The heterogeneity was low-o-moderate despite a lack of head-to-head comparison of some treatment regimens.

### Network Meta-Analysis

**Figure 2:**
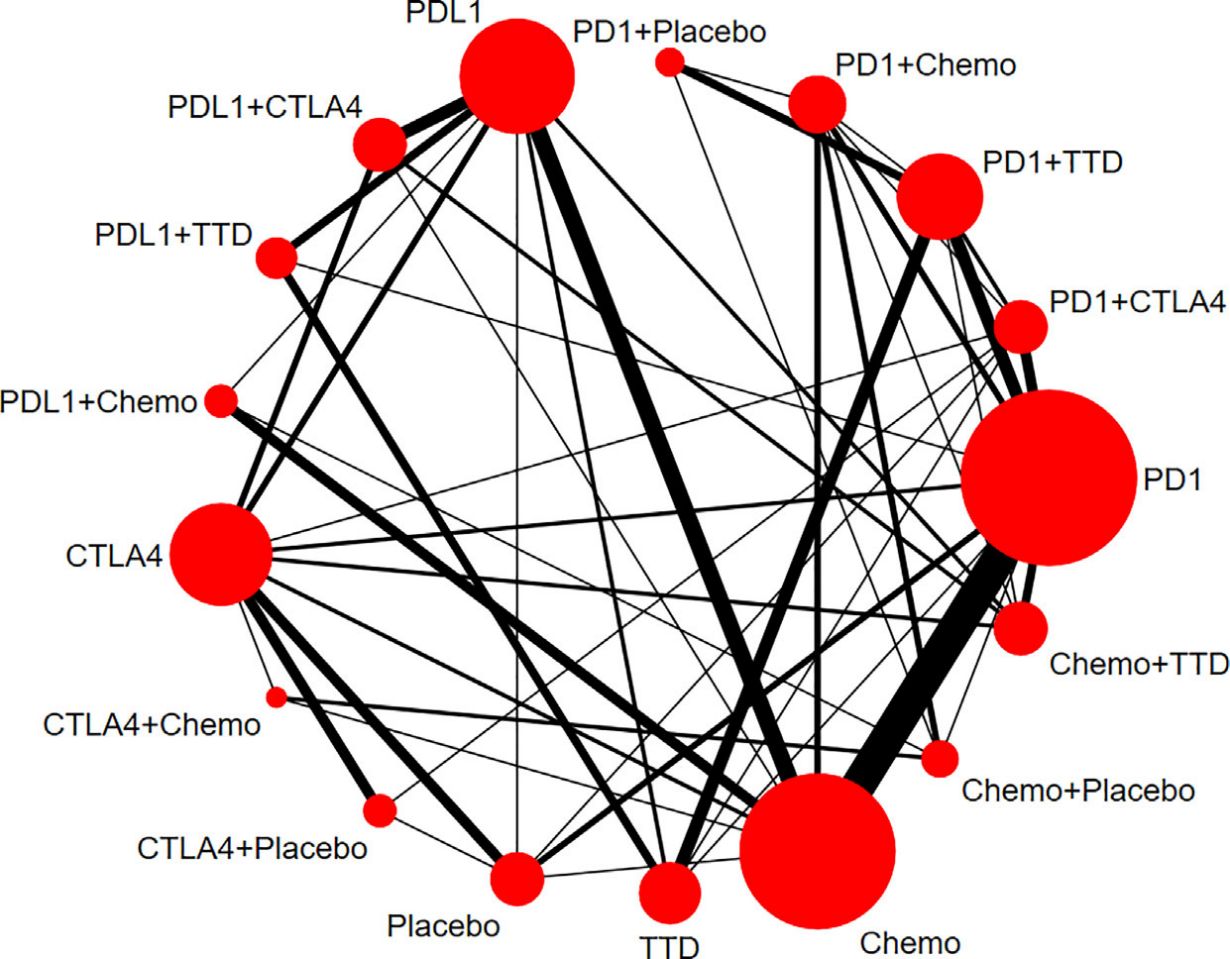
Network plots for renal adverse events. Nodes indicate the classes which are evaluated in clinical trials. Lines represent head-to-head comparisons of the two treatment regimens indicated by the connected nodes. The thickness of lines is weighted according to the number of trials comparing the two connected treatment regimens. The size of the node is proportional to the number of trials evaluating the treatment. TTD, targeted therapy drug; Chemo, chemotherapy; PD-1, programmed cell death 1; PD-L1, programmed cell death ligand 1; CTLA4, cytotoxic T-lymphocyte antigen 4.

**Figure 2** shows the network of all comparisons for RAEs. The results of the network meta-analysis in RAEs are given in **Table 1**. Moreover, we analyzed secondary outcomes to have a comprehensive understanding of the toxicity of different treatment regimens in terms of grade 3–5 RAEs, AKI, and grade 3–5 AKI. The results are shown in **Supplementary Figure 4** and **Supplementary Table 6**.

**Table 1:** Network estimates of treatment comparisons for RAEs and grade 3-5 RAEs.

**PD-1**	1.03 (0.53-2.02)	0.61 (0.29-1.21)	**0.54 (0.28-0.95)**	0.87 (0.47-1.62)	1.24 (0.46-3.43)	1.46 (0.68-3.41)	0.54 (0.21-1.28)	**0.33 (0.14-0.77)**	0.48 (0.17-1.27)	1.80 (0.95-3.53)	0.69 (0.40-1.13)	0.69 (0.30-1.58)	**3.10 (1.21-9.75)**
**0.62 (0.39-0.98)**	**PD1+CTLA4**	0.59 (0.24-1.37)	0.52 (0.21-1.19)	0.84 (0.36-1.99)	1.21 (0.38-3.88)	1.42 (0.56-3.94)	0.52 (0.17-1.51)	**0.32 (0.12-0.87)**	0.46 (0.14-1.48)	1.75 (0.78-3.99)	0.67 (0.29-1.45)	0.67 (0.24-1.86)	**3.02 (1.00-10.8)**
**0.57 (0.36-0.88)**	0.91 (0.54-1.55)	**PD1+TTD**	0.89 (0.37-2.05)	1.45 (0.60-3.54)	2.04 (0.66-6.87)	2.43 (0.93-6.90)	0.89 (0.30-2.64)	0.55 (0.19-1.58)	0.78 (0.24-2.59)	**2.97 (1.38-6.89)**	1.14 (0.49-2.57)	1.15 (0.41-3.14)	**5.13 (1.63-19.2)**
**0.37 (0.23-0.62)**	0.60 (0.32-1.14)	0.66 (0.36-1.22)	**PD1+Chemo**	1.62 (0.77-3.58)	2.31 (0.80-7.16)	**2.72 (1.10-7.87)**	1.00 (0.38-2.65)	0.61 (0.24-1.67)	0.88 (0.32-2.56)	**3.34 (1.49-8.25)**	1.29 (0.69-2.37)	1.29 (0.53-3.25)	**5.84 (1.98-20.7)**
0.77 (0.47-1.26)	1.24 (0.67-2.32)	1.36 (0.75-2.48)	**2.08 (1.09-3.92)**	**PDL1**	1.42 (0.57-3.71)	1.68 (0.75-4.10)	0.62 (0.24-1.51)	**0.38 (0.16-0.91)**	0.55 (0.19-1.56)	2.06 (0.96-4.61)	0.80 (0.44-1.32)	0.79 (0.32-1.96)	**3.54 (1.31-12.2)**
0.91 (0.46-1.82)	1.46 (0.68-3.24)	1.60 (0.75-3.53)	**2.44 (1.11-5.41)**	1.18 (0.63-2.24)	**PDL1+CTLA4**	1.18 (0.36-4.06)	0.43 (0.12-1.42)	**0.26 (0.09-0.75)**	0.38 (0.10-1.40)	1.45 (0.46-4.53)	0.56 (0.20-1.40)	0.56 (0.20-1.66)	2.51 (0.72-10.6)
0.84 (0.43-1.61)	1.34 (0.64-2.82)	1.47 (0.73-2.96)	2.24 (0.99-4.92)	1.08 (0.54-2.12)	0.92 (0.38-2.16)	**PDL1+TTD**	0.37 (0.11-1.09)	**0.22 (0.07-0.66)**	0.32 (0.09-1.07)	1.23 (0.56-2.55)	0.47 (0.18-1.06)	0.47 (0.15-1.39)	2.12 (0.63-8.17)
**0.40 (0.20-0.80)**	0.64 (0.29-1.45)	0.71 (0.32-1.57)	1.08 (0.49-2.33)	0.52 (0.25-1.09)	0.44 (0.18-1.06)	0.48 (0.20-1.21)	**PDL1+Chemo**	0.61 (0.20-1.97)	0.88 (0.27-2.95)	**3.35 (1.20-10.1)**	1.29 (0.57-2.80)	1.29 (0.41-4.03)	**5.81 (1.73-23.6)**
**0.51(0.29-0.91)**	0.82 (0.43-1.58)	0.91 (0.46-1.79)	1.38 (0.67-2.79)	0.66 (0.34-1.27)	0.56 (0.26-1.19)	0.61 (0.27-1.41)	1.28 (0.54-2.96)	**CTLA4**	1.44 (0.44-4.66)	**5.48 (2.00-15.5)**	2.09 (0.85-4.96)	2.09 (0.78-5.72)	**9.44 (3.66-29.7)**
0.47 (0.19-1.17)	0.76 (0.28-2.04)	0.83 (0.31-2.23)	1.27 (0.48-3.27)	0.61 (0.23-1.61)	0.52 (0.17-1.50)	0.57 (0.19-1.71)	1.17 (0.40-3.39)	0.92 (0.35-2.42)	**CTLA4+Chemo**	**3.81 (1.21-12.3)**	1.46 (0.53-3.73)	1.46 (0.43-4.93)	**6.57 (1.81-28.9)**
0.86 (0.54-1.38)	1.37 (0.80-2.40)	1.51 (0.97-2.39)	**2.31 (1.20-4.37)**	1.11 (0.62-1.99)	0.94 (0.43-2.02)	1.03 (0.56-1.90)	2.14 (0.96-4.74)	1.67 (0.84-3.39)	1.82 (0.67-5.00)	**TTD**	**0.38 (0.17-0.79)**	0.39 (0.14-1.04)	1.73 (0.57-6.01)
0.74 (0.52-1.06)	1.19 (0.69-2.07)	1.31 (0.78-2.23)	**2.00 (1.16-3.40)**	0.96 (0.61-1.50)	0.82 (0.42-1.55)	**0.28 (0.12-0.59)**	**1.85 (1.00-3.42)**	1.45 (0.79-2.68)	1.58 (0.64-3.95)	0.87 (0.50-1.49)	**Chemo**	1.00(0.43-2.43)	**4.52(1.68-15.4)**
**0.48 (0.27-0.87)**	0.77 (0.38-1.56)	0.85 (0.43-1.68)	1.30 (0.65-2.52)	0.63 (0.32-1.20)	0.53 (0.24-1.14)	0.58 (0.25-1.33)	1.20 (0.51-2.82)	0.94 (0.46-1.94)	1.02 (0.36-2.90)	0.56 (0.28-1.13)	0.65 (0.35-1.21)	**Chemo+TTD**	**4.52 (1.39-16.5)**
**2.70 (1.33-5.82)**	**4.33 (2.03-9.80)**	**4.77 (2.14-11.2)**	**7.25 (3.13-17.5)**	**3.49 (1.58-8.17)**	**2.96 (1.19-7.68)**	**3.24 (1.27-8.70)**	**6.73 (2.59-18.3)**	**5.26 (2.59-11.5)**	**5.75 (1.91-18.0)**	**3.15 (1.39-7.46)**	**3.63 (1.71-8.20)**	**5.61 (2.35-14.1)**	**Placebo**

Network estimates of treatment comparisons for RAEs (on the lower triangle) and grade 3-5 RAEs (on the upper triangle). The summary estimates are risk ratios (RRs) and 95% confidence intervals. For RAEs, the column-defining treatment is compared to the row-defining treatment, and RRs < 1 favor the column-defining treatment. For grade 3-5 RAEs, the row-defining treatment is compared to the column-defining treatment, and RRs < 1 favor the row-defining treatment.

Significant results are in bold.

RAEs, renal adverse events; PD-1, programmed cell death 1; PD-L1, programmed cell death ligand 1; CTLA4, cytotoxic T-lymphocyte antigen 4; Chemo, Chemotherapy; TTD, Targeted therapy drug.

#### RAEs

Compared with placebo, all other treatment regimens significantly increased the risk of RAEs, with effect sizes ranging from 2.70 (95%CI: 1.33-5.82) for anti-PD-1 to 7.25 (95%CI: 3.13-17.5) for anti-PD-1 plus chemotherapy. With regard to ICI monotherapy, anti-PD-1 (RR: 0.51, 95%CI: 0.29-0.91) was significantly safer than anti-CTLA-4; however, there was no significant difference between anti-PD-1 and anti-PD-L1 with respect to safety. Anti-PD-1 plus anti-CTLA-4 (RR: 1.62, 95%CI: 1.05-2.56), anti-PD-1 plus chemotherapy (RR: 2.50, 95%CI: 1.25-0.50), and anti-PD-1 plus targeted therapy (RR: 1.75, 95%CI: 1.14-2.78) all displayed higher risk than anti-PD-1 alone. Further, anti-PD-1 plus chemotherapy (RR: 2.00, 95%CI: 1.16-3.40) and anti-PD-L1 plus chemotherapy (RR: 1.85, 95%CI: 1.00-3.42) were associated with higher risk than chemotherapy. There were no significant differences between anti-PD-1 plus targeted therapy or anti-PD-L1 plus targeted therapy and targeted therapy.

**Figure 3:**
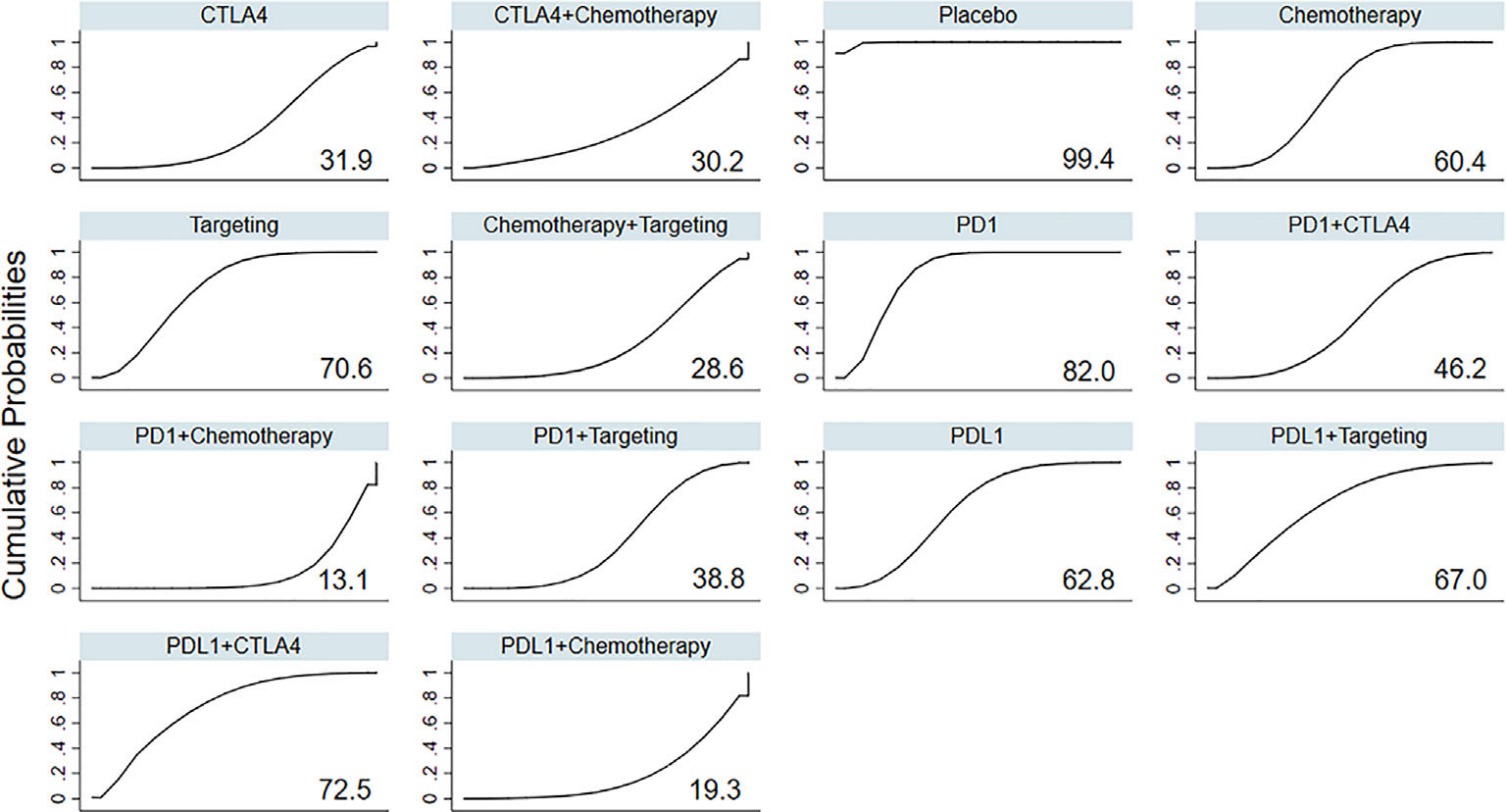
Rankings of SUCRA for the risk of RAEs. SUCRA, surface under the cumulative ranking; RAEs, renal adverse events; PD-1, programmed cell death 1; PD-L1, programmed cell death ligand 1; CTLA4, cytotoxic T-lymphocyte antigen 4.

Based on the ranking curves (**Figure 3** and **Supplementary Figure 5**), ICIs plus chemotherapy seemed to be the most toxic treatment regimen in terms of RAEs and had the worst rank, whereas anti-PD-1 monotherapy seemed to be the least toxic one, followed by anti-PD-L1 plus anti-CTLA-4.

#### Grade 3–5 RAEs

All treatment regimens enhanced the risk of grade 3**–**5 RAEs in varying degrees compared with placebo, except anti-PD-L1 plus anti-CTLA-4 and anti-PD-L1 plus targeted therapy. Anti-CTLA-4 showed significantly higher toxicity than anti-PD-1 (RR: 0.33, 95%CI: 0.14-0.77), anti-PD-L1 (RR: 0.38, 95%CI: 0.16-0.91), anti-PD-1 plus anti-CTLA-4 (RR: 0.32, 95%CI: 0.12-0.87), and anti-PD-L1 plus anti-CTLA-4 (RR: 0.26, 95%CI: 0.09-0.75). Furthermore, anti-PD-1 plus targeted therapy, anti-PD-1 plus chemotherapy, anti-PD-L1 plus chemotherapy, anti-CTLA-4, anti-CTLA-4 plus chemotherapy, and chemotherapy showed significantly higher risk than targeted therapy. The estimated effects were not significant between chemotherapy and ICIs plus chemotherapy.

Anti-CTLA-4 appeared to be the most toxic treatment regimen with an RR of 9.44 (95% CI: 3.66–29.7), whereas targeted therapy appeared to be the least toxic regimen with the best rank (**Supplementary Figures 5** and **7**).

#### Acute Kidney Injury

Apart from anti-PD-1 plus anti-CTLA-4, anti-PD-L1 plus targeted therapy, and targeted therapy, all other treatment regimens showed increased risk of AKI compared with placebo. The toxic effects of ICI monotherapy and combination therapy were not significantly different, except that anti-PD-1 plus chemotherapy had a significantly higher risk of AKI than the anti-PD-1 regimen. In addition, anti-PD-1, anti-PD-L1, anti-CTLA-4, chemotherapy, anti-PD-1 plus chemotherapy, anti-PD-1 plus targeted therapy, and chemotherapy plus targeted therapy all showed markedly higher risk of AKI than targeted therapy. ICIs plus chemotherapy seemed to be the most toxic treatment regimen, whereas targeted therapy seemed to be the least toxic one in terms of AKI (**Supplementary Figures 6** and **7**).

We had similar findings in the grade 3–5 AKI to those in AKI. Apart from anti-PD-1 plus anti-CTLA-4, anti-PD-L1 plus anti-CTLA-4, anti-PD-L1 plus targeted therapy, and targeted therapy, all other treatment regimens showed increased risk of grade 3-5 AKI compared with placebo. Moreover, all other regimens except anti-PD-L1 plus anti-CTLA-4 had significantly higher risk of grade 3-5 AKI than targeted therapy. SUCRAs and rankings were similar for AKI and grade 3–5 AKI (**Supplementary Figures 6** and **7**).

### Transitivity, Inconsistency, Heterogeneity, and Sensitivity Analysis

The random consistency model had the lowest DIC value than the other three models, which manifested that it was the preferred model with a better trade-off between model fit and complexity (**Supplementary Table 7**). Assessment of transitivity for RAEs indicated that the median age, sex ratio, sample size, and trial start year across treatment comparisons were relatively similar and thus no threats to the transitivity assumption were identified (**Supplementary Figure 8**). The “design-by-treatment” interaction models found no evidence for global inconsistency for all outcomes. Concerning the local inconsistency, the loop-specific method (**Supplementary Figure 9**) and node-split model (**Supplementary Table 8**) revealed no significant discrepancy between the direct and indirect comparisons, except for one comparison (placebo *vs* anti-PD-L1, p=0.014). The median heterogeneity (τ²) was estimated at 0.20 (95%CI: 0.09–0.40) for RAEs, 0.12 (95%CI: 0.00–0.57) for grade 3–5 RAEs, 0.12 (95%CI: 0.00–0.64) for AKI, and 0.17 (95%CI: 0.00–1.00) for grade 3–5 AKI, all suggesting low heterogeneity. Meta-regression analysis for RAEs revealed that tumor types might be a source of heterogeneity (**Supplementary Table 9**). Thus, we performed a subgroup analysis based on tumor types, which showed that the distribution of SUCRA values remarkably varied across different cancers in terms of RAEs (**Supplementary Table 10** and **Supplementary Figure 10**). Thus, it is reasonable to infer that tumor type may be a source of heterogeneity, and therefore our findings may not directly apply to different kinds of tumors.

It was worth noting that the effects of three kinds of regimens—anti-PD-1 plus anti-CTLA-4, anti-PD-1 plus targeted therapy, and anti-CTLA-4—were not significant when compared to anti-PD-1 in certain sensitivity analyses (**Supplementary Table 11**). Reduced sample size may be the reason for the statistically non-significant RRs and wide confidence intervals. However, there were no obvious changes in the most and least toxic treatment regimens. Hence, the overall results were relatively stable and robust.

## Discussion

This network meta-analysis included 95 RCTs involving 40,552 patients and compared 14 treatment regimens. In this study, we explored the RAEs in patients with ICIs, which manifest not only as AKI but also as other types of renal damage that may not meet the criteria of AKI. Both ICI-based treatment regimens and traditional cancer therapies showed significantly higher risk of RAEs than placebo. With regard to ICI monotherapy, anti-CTLA-4 showed remarkably higher risk of RAEs than anti-PD-1 and significantly greater risk of grade 3–5 RAEs than anti-PD-1 and anti-PD-L1 regimens. We did not find significant differences between ICI monotherapy and traditional cancer therapy in terms of RAEs. However, chemotherapy and ICI monotherapy both incurred significantly higher odds of AKI than targeted therapy. Anti-CTLA-4 plus anti-PD-1 were associated with higher risk of RAEs than anti-PD-1. The difference was not significant between other dual ICI regimens and ICI monotherapy in terms of RAEs and AKI. In addition, ICI plus chemotherapy showed increased risk of both RAEs and AKI to varying degrees than ICIs monotherapy, chemotherapy, and targeted therapy.

Our study found that the overall incidence of RAEs, grade 3–5 RAEs, AKI, and grade 3–5 AKI was 4.3%, 1.2%, 1.3%, and 0.8%, respectively. However, the incidence of AKI was found to be 15.5–17% in patients receiving ICIs in some retrospective studies (31–34). The main reason for this difference may be because of the different samples of patients enrolled. Unlike the general hospital populations in retrospective studies, patients in RCTs are always in a better condition, usually with an Eastern Cooperative Oncology Group (ECOG) performance status of 0 or 1. In addition, patients in RCTs are always highly selected. For example, patients with active brain metastases, autoimmune disease, or human immunodeficiency virus infection were excluded in some RCTs. In addition, the incidence of AKI was probably overestimated in retrospective studies as AKI due to other reasons (e.g., hemodynamic, sepsis-related, or obstructive AKI) may also be included. Whereas by including RCTs only, our study focused on ICI–related AKI. Therefore, our meta-analysis may reflect the incidence of AKI with less bias.

Our study suggested that among ICI monotherapy, anti-CTLA-4 showed remarkably higher risk of RAEs than anti-PD-1 and significantly increased risk of grade 3–5 RAEs than the anti-PD-1 and anti-PD-L1 regimens. These differences in the risk of RAEs may be attributed to the individual mechanisms of action of each medication. Although both anti-CTLA-4 and anti-PD-1 can restore antitumor immunity, they function in distinct ways. CTLA-4 exerts its regulatory effect during the early phase of the immune response within lymphoid organs. PD-1, on the other hand, exerts its regulatory effect later in the course of T cell activation within peripheral tissues (6). PD-L1, the ligand of PD-1, is expressed in the kidney tubules. Anti-PD-1 is speculated to alter T cell immune tolerance against endogenous antigens in the kidney or concomitant drugs that might trigger AIN (20, 35). The upstream and less specific effect of anti-CTLA-4 may be responsible for higher toxicity compared to anti-PD-1.

Previously, a study concluded that AKI occurred more frequently in patients who received dual ICI therapy than in patients who received ICI monotherapy (20). However, as an increasing number of ICIs are approved by the FDA in a larger sample of patients, the risk of dual ICI therapy needs to be re-evaluated. Recently, several retrospective cohort studies in different centers have found that ICI combination therapy was not a risk factor for AKI (31, 34, 36). Including the most recent studies, our study found that anti-CTLA-4 plus anti-PD-1 was associated with higher risk of RAEs than anti-PD-1. However, this difference was not significant between anti-PD-1 plus anti-CTLA-4 and anti-PD-1 in terms of AKI. More comprehensive studies and further analyses are needed to determine the incidence of RAEs of dual ICI therapies. Our study implied that ICIs plus chemotherapy is the most toxic treatment regimen in terms of RAEs, AKI, and grade 3–5 AKI. One reason for this may be the different mechanism of ICIs and chemotherapy. Conventional chemotherapeutic drugs can induce AKI by injuring multiple renal compartments including renal microvasculature, glomerulus, renal interstitium, and tubular segments (37). Drugs such as platinum-containing regimens and pemetrexed can cause direct cellular toxicity owing to their excretion through tubular cells, development of inflammation and oxidative stress, and activation of apoptotic and necrotic signaling pathways (38). Another reason may be the synergistic effects of ICIs and chemotherapy. Chemotherapy was reported to enhance the expression of PD-L1, thus improving the antitumor activity of ICIs when combining immunotherapy with chemotherapy (39, 40). In our meta-analysis, dual ICI therapy and ICIs plus targeted therapy seemed to be less toxic than ICIs plus chemotherapy. Therefore, they may be considered as a priority for patients who showed no response to ICI monotherapy or with poor kidney function.

The incidence of AKI was reported to be associated with increased mortality and morbidity and limited use of treatment regimens in patients with cancer. Severe AKI is also known to be associated with longer length of hospital stay and higher daily costs in hospital (41–43). Thus, clinicians must tailor treatment options with a better trade-off between benefits and toxicity, especially in patients with a high risk of RAEs. Our analysis suggested that anti-PD-1 seemed to be the least toxic regimen in terms of RAEs, making it a suitable choice of treatment. To our knowledge, this is the largest and most comprehensive study to compare the risk of RAEs among ICI-based therapy. Furthermore, we provide the incidence and risks of RAEs of ICIs combined with traditional cancer therapy which is still poorly understood. Our meta-analysis has some limitations. First, it was conducted at the study level rather than the individual patient data level, as potentially important variables at the patient level such as background nephropathy were not imported in the analysis. RCTs with more comprehensive data are needed to nullify these factors. Second, although the results remained stable after the meta-regression of cancer types, subgroup analysis suggested that the risk of RAEs varied remarkably across different cancer types that might be attributed to the property intrinsic to specific cancer types. The results could be misinterpreted when evaluating the possible reasons for renal impairment in such cases (disease-related *vs.* treatment-related). Input from other specialties (e.g., nephrologists and urologists) is of paramount significance in the individual management of such cases. Third, we performed analysis on different ICI classes instead of individual ICIs and particular doses, which might lead to variations in study outcomes. Similarly, different chemotherapeutic or targeted drugs with different incidences of RAEs were defined as one class that might be a source of heterogeneity. Nonetheless, differentiating treatment regimens based on individual drugs and particular dosage was not feasible due to limited samples. Finally, because patients with end stage renal disease (ESRD) are usually excluded from clinical trials, these results cannot be generalized to all patients. More data and further analysis in ESRD patients are necessary for a more in-depth understanding of the application of ICIs.

### Conclusion

Our network meta-analysis has highlighted the risks of RAEs between ICI monotherapy, ICI combination therapy, and traditional cancer therapy and provided oncologists with a nephrology perspective of choosing different treatment regimens. Further studies are needed for a better understanding of RAEs among patients with different cancer types, using different ICI doses and with different kidney function.

## Data Availability Statement

The original contributions presented in the study are included in the article/**Supplementary Material**. Further inquiries can be directed to the corresponding author.

## Author Contributions

Concept and design: KL and HM. Extraction and collection of data: ZQ, KL, and XX. Statistical analysis: KL, TL, and YG. Drafting and revision of manuscript: KL, ZQ, and HM. Supervision: HM and CX. Final approval of manuscript: KL, ZQ, XX, TL, YG, HM, and CX. All authors contributed to the article and approved the submitted version.

## Funding

The present study was supported by the Priority Academic Program Development of Jiangsu Higher Education Institutions (CN), the National Natural Science Foundation General Project (81970639), the Scientific Research Project of Jiangsu Provincial Health and Wellness Commission (H2017023), and the Six Talent Peaks Project in Jiangsu Province (grant no. WSN-056). The funders had no role in the design of the study and collection, analysis, and interpretation of data and in writing the manuscript.

## Conflict of Interest

The authors declare that the research was conducted in the absence of any commercial or financial relationships that could be construed as a potential conflict of interest.

## Abbreviations

AKI, acute kidney injury; CKD, chronic kidney disease; CTCAE, Common Terminology Criteria for Adverse Events; CTLA-4, cytotoxic T-lymphocyte antigen 4; DIC, deviance information criterion; eGFR, estimated glomerular filtration rate; ESRD, end stage renal disease; FDA, Food and Drug Administration; ICIs, immune checkpoint inhibitors; ICI-AKI, immune checkpoint inhibitor-associated AKI; irAEs, immune-related adverse events; NSCLC, non-small cell lung cancer; PD-1, programmed cell death 1; PD-L1, programmed cell death ligand 1; PRISMA, Preferred Reporting Items for Systematic Reviews and Meta-Analyses; RAEs, renal adverse events; RCTs, randomized clinical trials; RR, risk ratio; SUCRA, surface under the cumulative ranking; TIN, tubulointerstitial nephritis; TRAEs, treatment-related adverse events.
