# Supplementary material for: Comparative Risk of Renal Adverse Events in Patients Receiving Immune Checkpoint Inhibitors: A Bayesian Network Meta-Analysis
Source: Front Oncol. 2021 Jun 16;11:662731. doi: 10.3389/fonc.2021.662731 (PMC8242344; doi:10.3389/fonc.2021.662731)
Supplement: Supplementary file 1 [file DataSheet_1.pdf]

Comparative risk of renal adverse events in patients receiving immune checkpoint inhibitors: A Bayesian Network Meta-analysis

Supplemental materials

Supplemental Figure 1. Incidence of RAEs among different kinds of treatment regimens 1

Supplemental Figure 2. Risk of bias assessments of all included trials 2

Supplemental Figure 3. Comparison-adjusted funnel plots of publication bias test 3

Supplemental Figure 4. Network plots 4

Supplemental Figure 5. Ranking probabilities curves for RAEs 5

Supplemental Figure 6. Ranking probabilities curves for AKI 6

Supplemental Figure 7. Rankings of SUCRA 7

Supplemental Figure 8. Assessment of transitivity 8

Supplemental Figure 9. Evaluation of local inconsistency using Loop-specific method 9

Supplemental Figure 10. The distribution of SUCRA values stratified by cancer types 10

Table S1. PRISMA NMA Checklist 11

Table S2. Search strategy 15

Table S3. FDA-approved doses of immune checkpoint inhibitors 16

Table S4. Main characteristics of the trials included in this meta-analysis 17

Table S5. Results of traditional pairwise meta-analysis for RAEs 24

Table S6. Network estimates of treatment comparisons for AKI and grade 3-5 AKI 25

Table S7. Evaluation of the model fit 26

Table S8. Evaluation of local inconsistency using node-split model 27

Table S9. Subgroup analyses and meta-regression 28

Table S10. SUCRA values stratified by cancer types 29

Table S11. Sensitivity analyses 30

Supplemental Figure 1. Incidence of RAEs among different kinds of treatment regimens. *RAEs, renal adverse events; AKI, acute kidney injury; TTD, Targeted therapy drug; Chemo, Chemotherapy; PD-1, programmed cell death 1; PD-L1, programmed cell death ligand 1; CTLA4, cytotoxic T-lymphocyte antigen 4.*

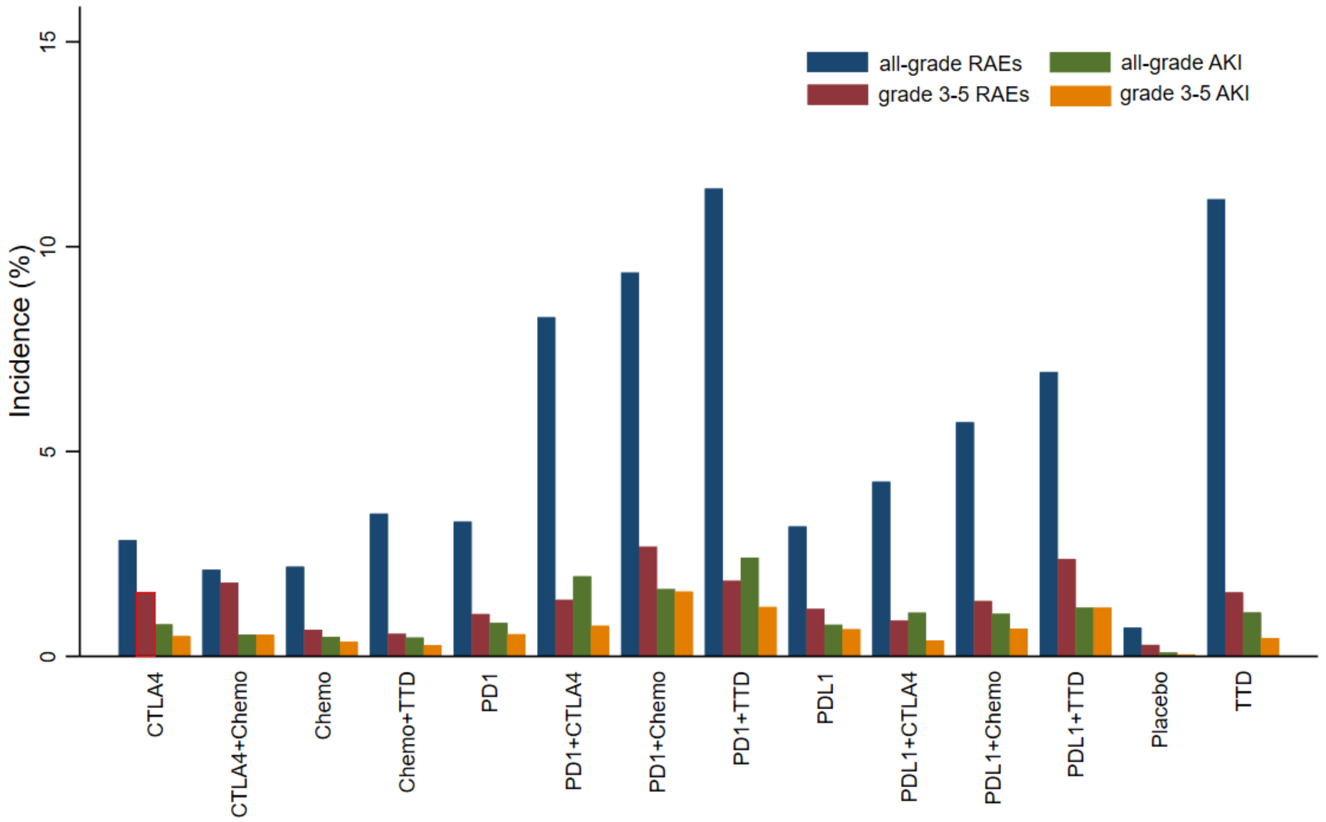

Supplemental Figure 2. Risk of bias assessments of all included trials. “+” low risk of bias; “?” unclear risk of bias; “-” high risk of bias.

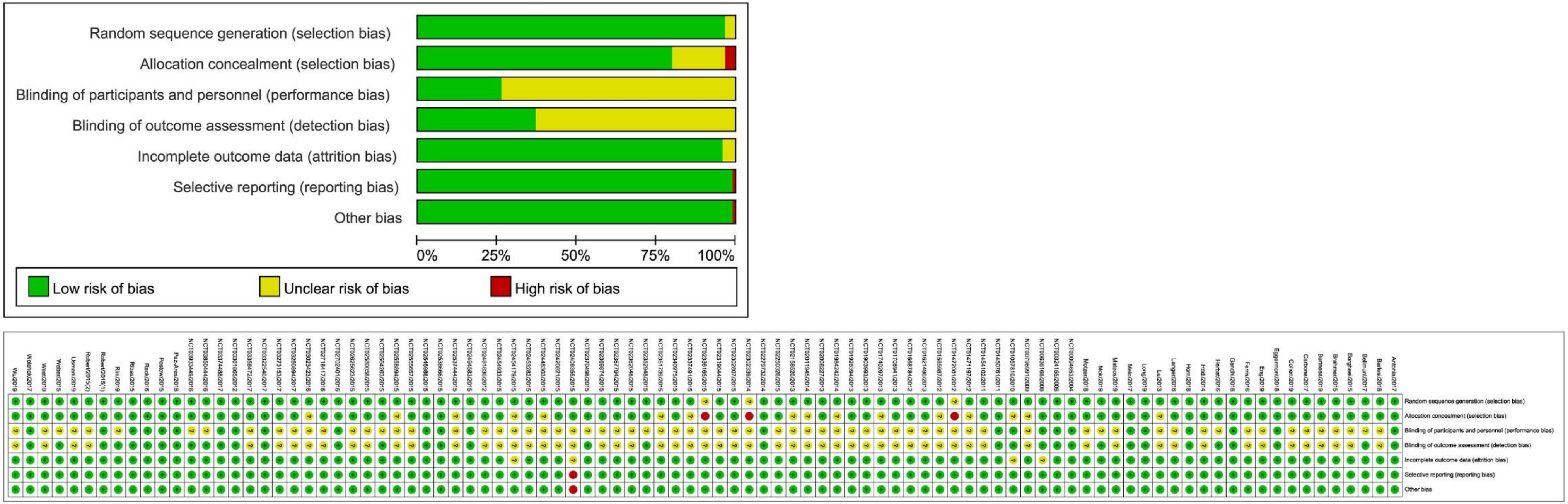

Supplemental Figure 3. Comparison-adjusted funnel plots of publication bias test for (A) RAEs, (B) grade 3-5 RAEs, (C) AKI, and (D) grade 3-5 AKI. *RAEs, renal adverse events; AKI, acute kidney injury.*

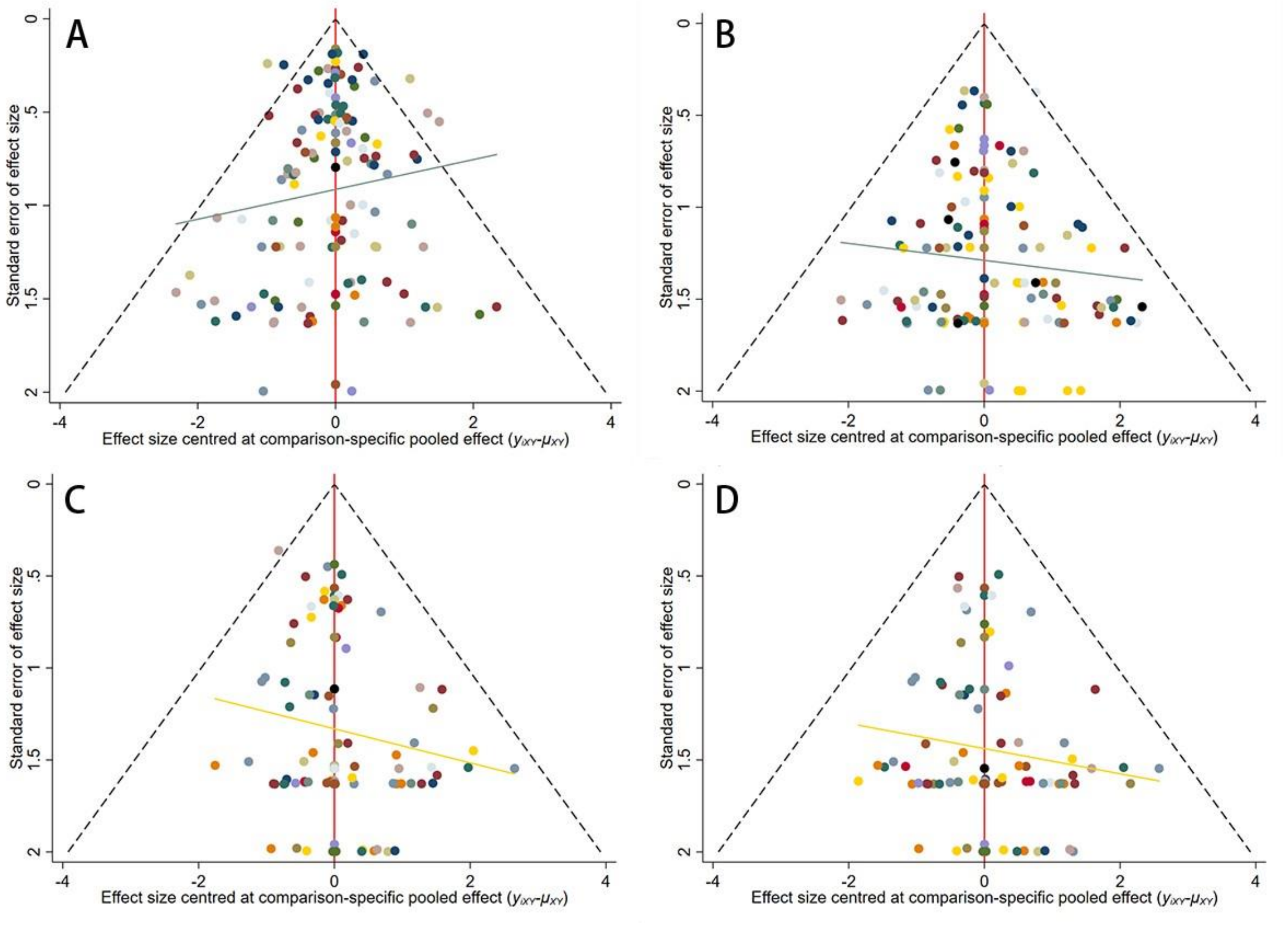

Supplemental Figure 4. Network plots for (A) grade 3-5 RAEs, (B) AKI, (C) grade 3-5 AKI, and RAEs in patients with (D) respiratory system cancer, (E) digestive system cancer, (F) urogenital system cancer (G) hematologic system cancer, and (H) skin cancer. Nodes indicate the classes which are evaluated in clinical trials. Lines represent head-to-head comparisons of the two treatment regimens indicated by the connected nodes. The thickness of lines is weighted according to the number of trials comparing the two connected treatment regimens. The size of the node is proportional to the number of trials evaluating the treatment. *RAEs, renal adverse events; AKI, acute kidney injury; TTD, Targeted therapy drug; Chemo, Chemotherapy; PD-1, programmed cell death 1; PD-L1, programmed cell death ligand 1; CTLA4, cytotoxic T-lymphocyte antigen 4.*

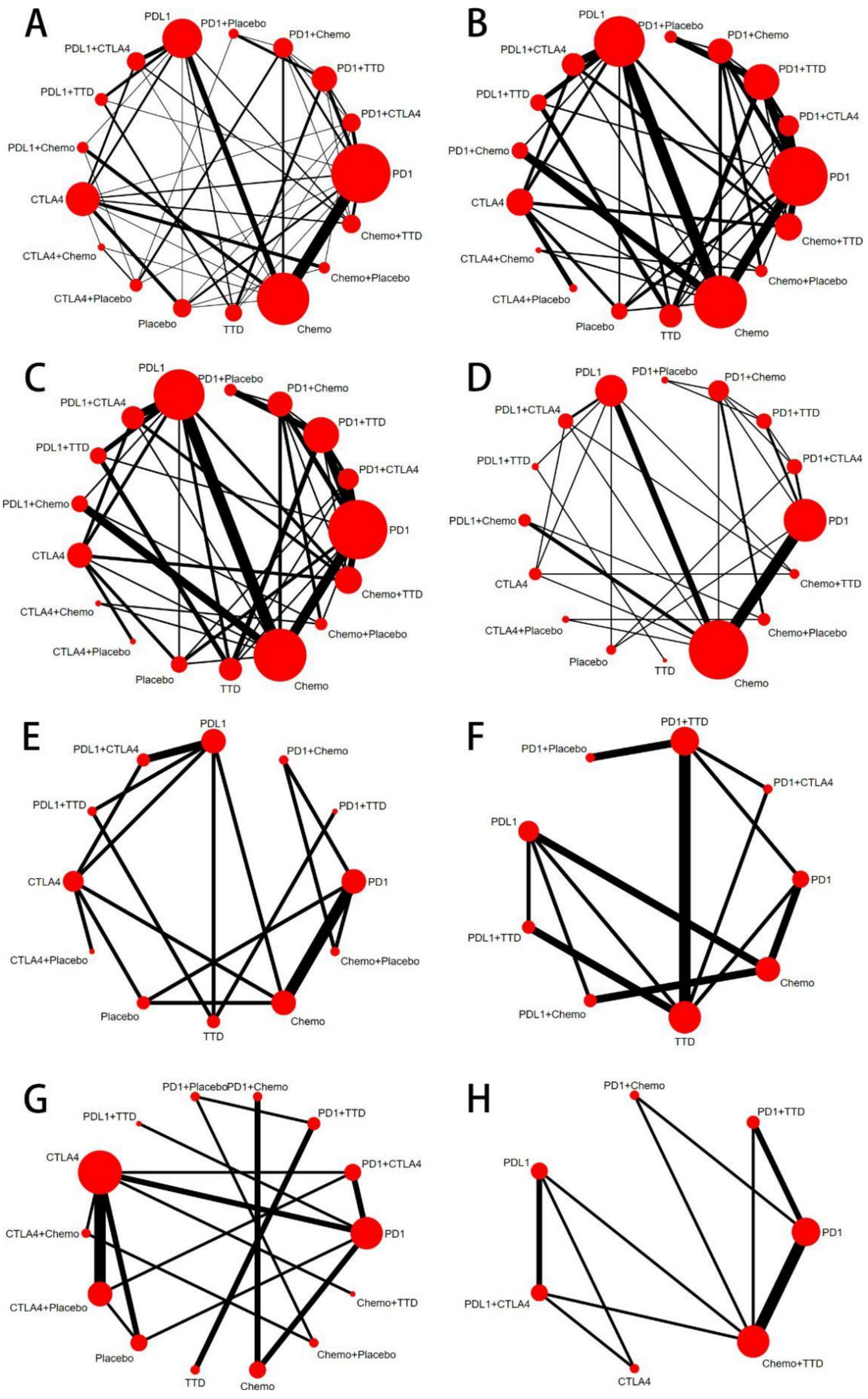

Supplemental Figure 5. Ranking probabilities curves for the risk of (A) RAEs, (B) grade 3-5 RAEs. The graphs display the distribution of probabilities of treatment ranking from best through worst for each outcome. The peak indicates the ranking with the highest probability for the corresponding treatment regimen. *RAEs, renal adverse events; PD-1, programmed cell death 1; PD-L1, programmed cell death ligand 1; CTLA4, cytotoxic T-lymphocyte antigen 4.*

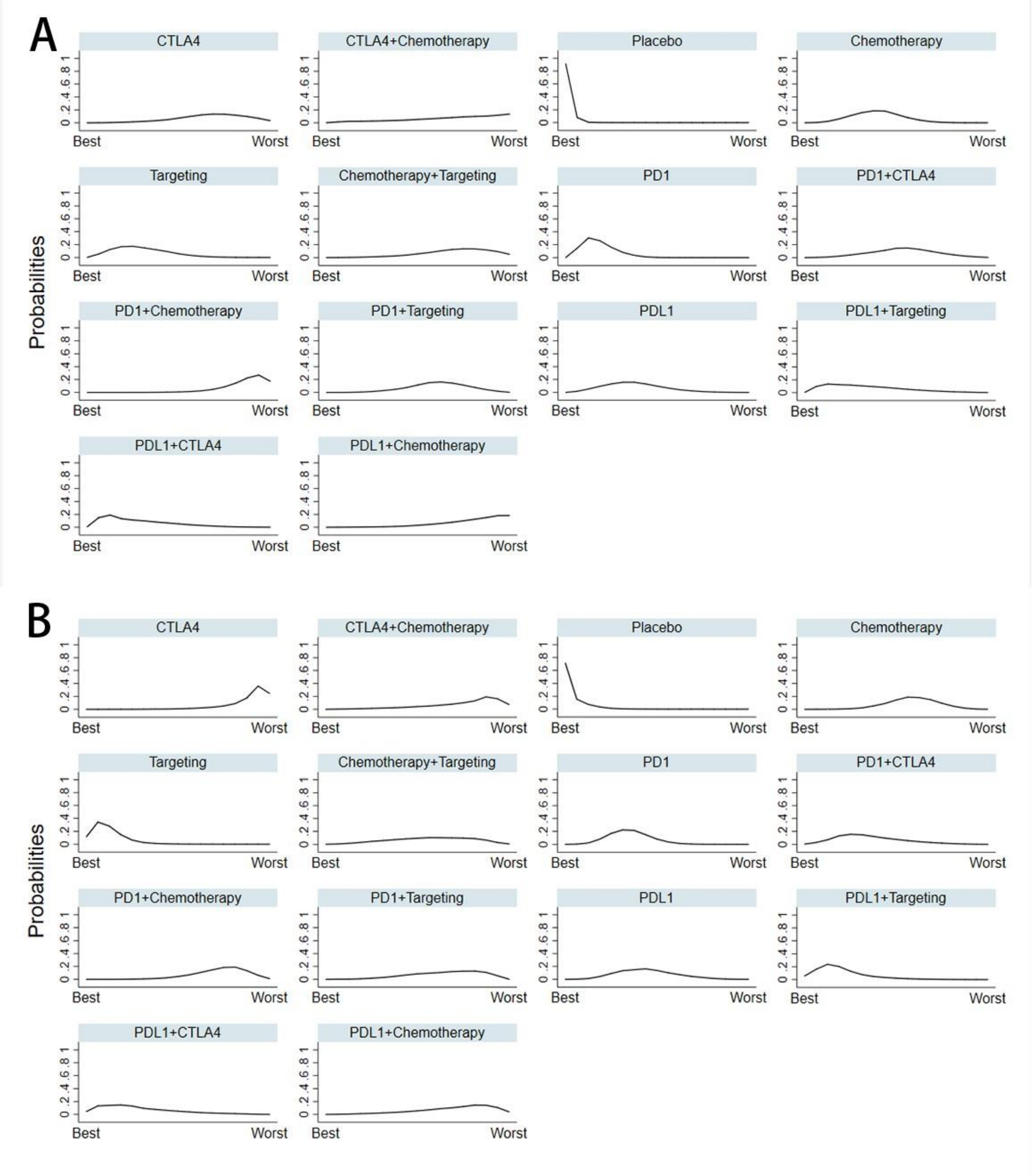

Supplemental Figure 6. Ranking probabilities curves for the risk of (A) AKI, (B) grade 3-5 AKI. The graphs display the distribution of probabilities of treatment ranking from best through worst for each outcome. The peak indicates the ranking with the highest probability for the corresponding treatment regimen. *AKI, acute kidney injury; PD-1, programmed cell death 1; PD-L1, programmed cell death ligand 1; CTLA4, cytotoxic T-lymphocyte antigen 4.*

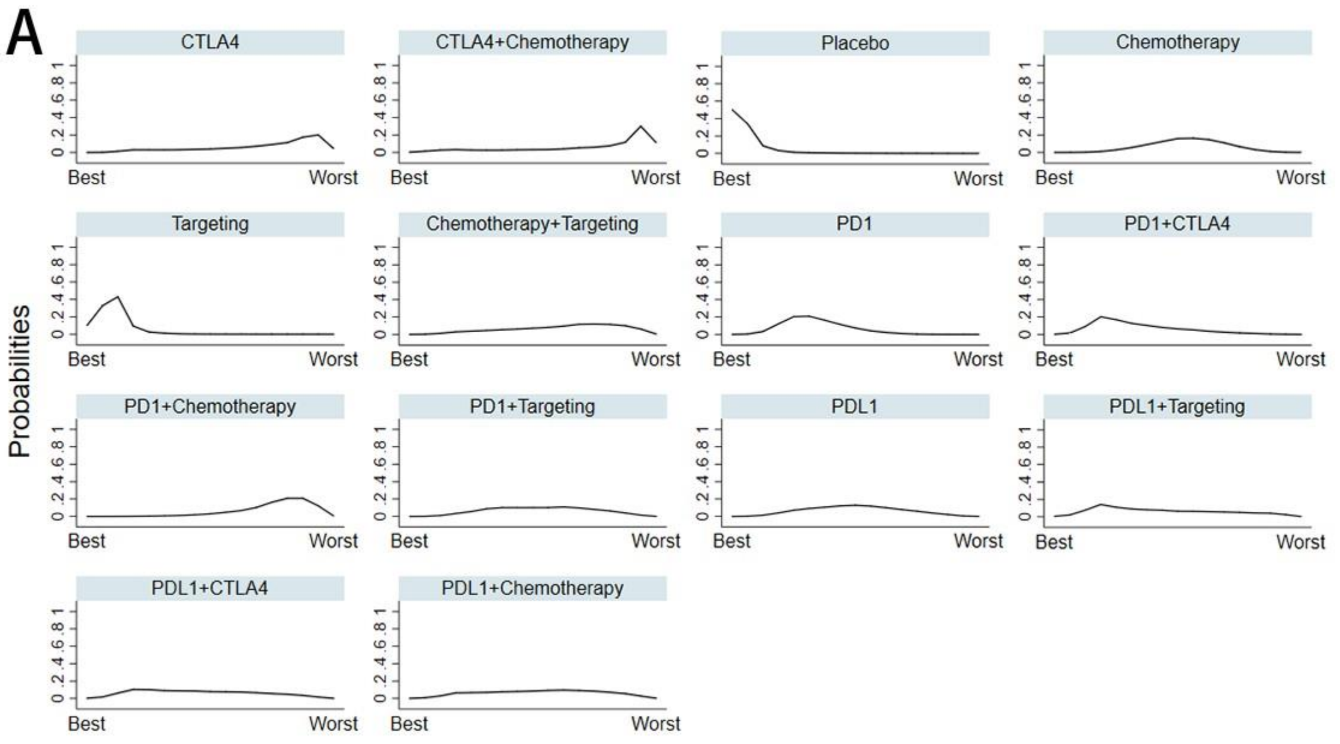

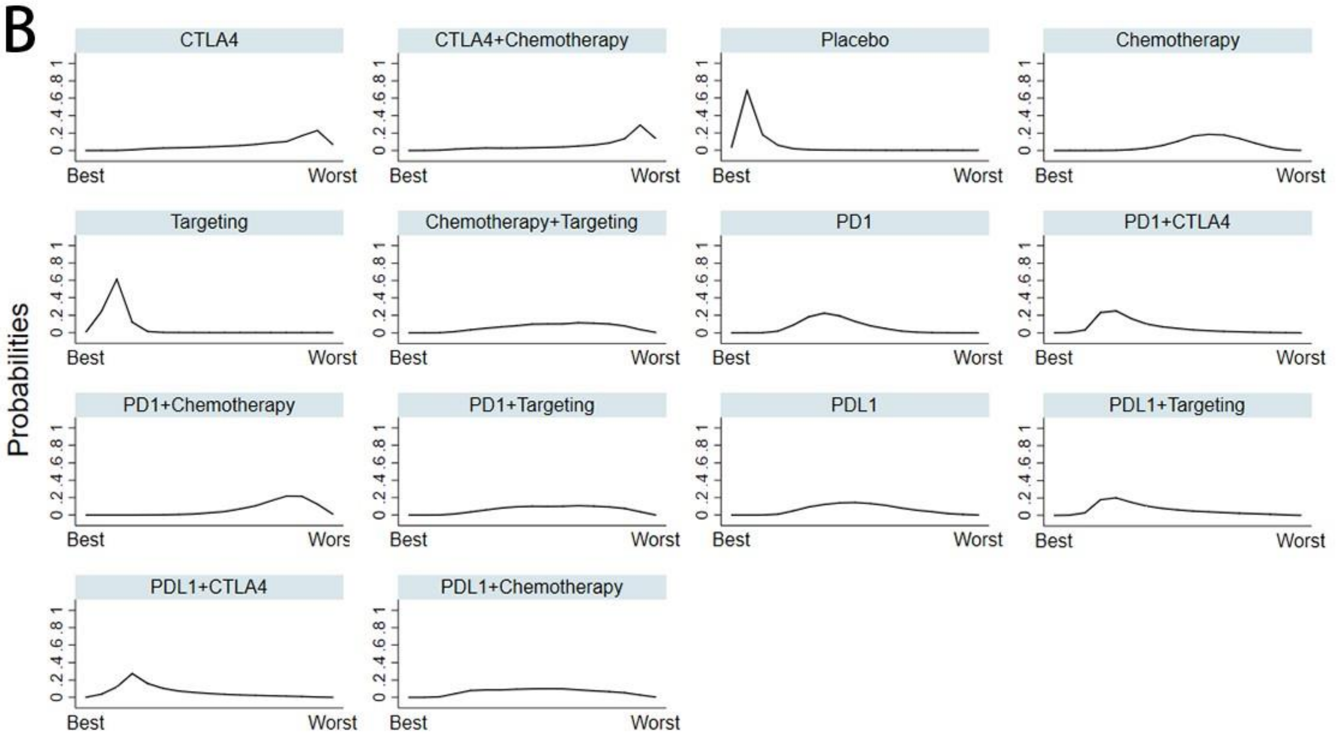

Supplemental Figure 7. Rankings of SUCRA for the risk of (A) grade 3-5 RAEs, (B) AKI, and (C) grade 3-5 AKI. *SUCRA, surface under the cumulative ranking; RAEs, renal adverse events; AKI, acute kidney injury. PD-1, programmed cell death 1; PD -L1, programmed cell death ligand 1; CTLA4, cytotoxic T-lymphocyte antigen 4.*

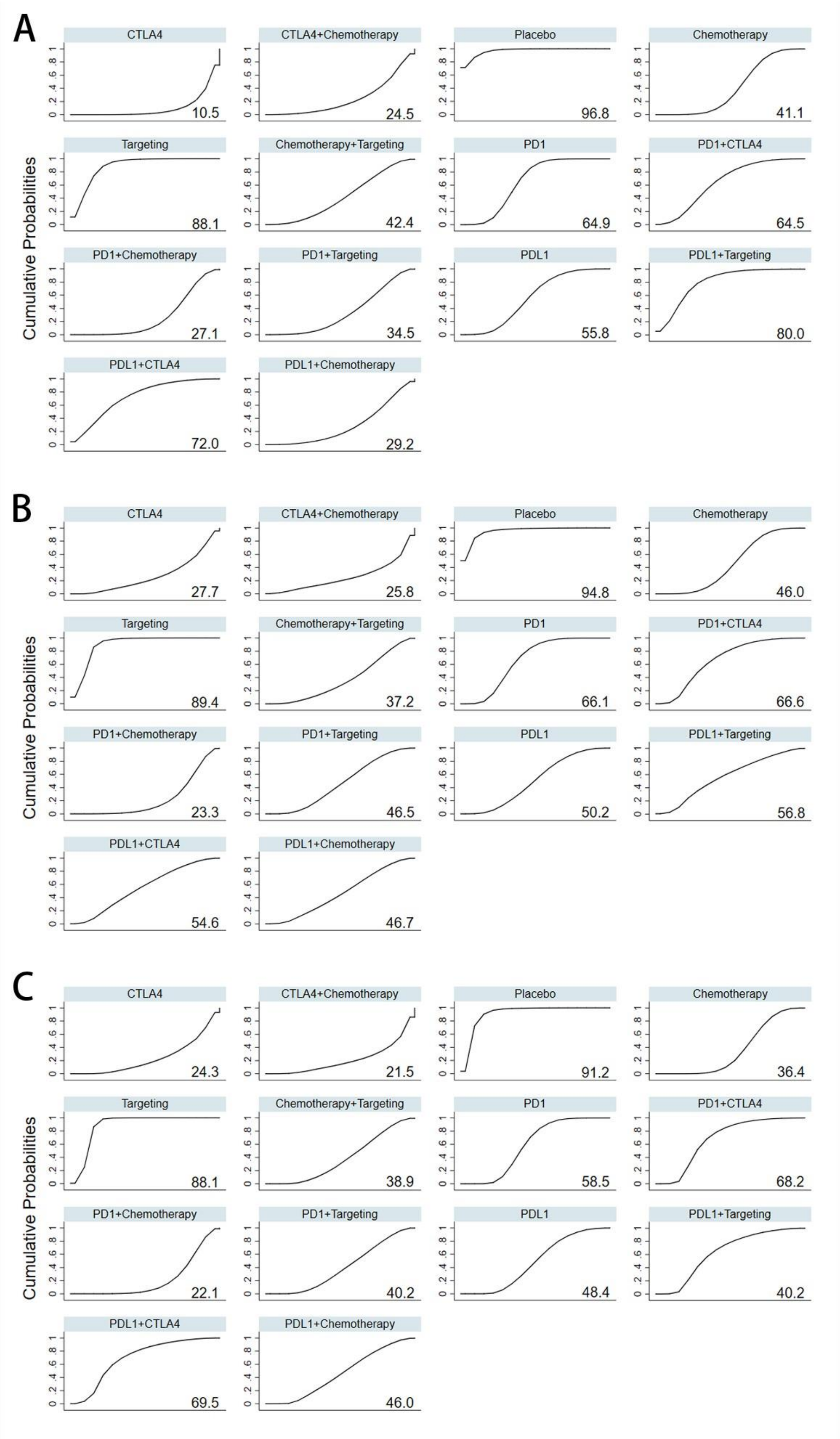

Supplemental Figure 8. Assessment of transitivity among all included trials. (A) Age, (B) Male, (C) Number of patients, and (D) Year. *TTD, Targeted therapy drug; Chemo, Chemotherapy; PD-1, programmed cell death 1; PD-L1, programmed cell death ligand 1; CTLA4, cytotoxic T-lymphocyte antigen 4.*

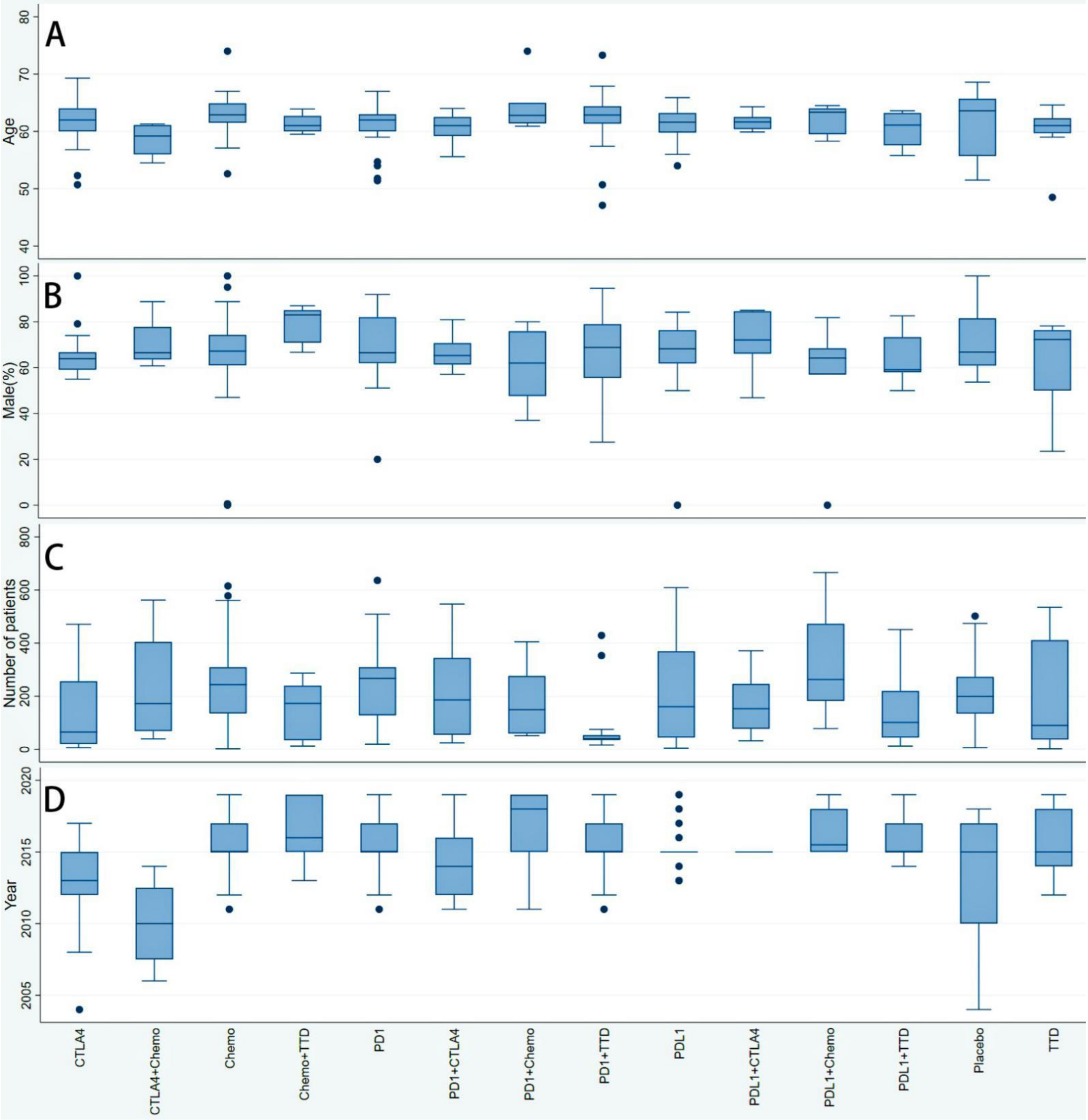

Supplemental Figure 9. Evaluation of local inconsistency for RAEs using Loop-specific method. Inconsistency refers to differences in effect estimates between direct and indirect comparisons, which could be evaluated when 3 treatments are connected within a loop. For each closed loop, we estimated the absolute difference between the direct and indirect comparisons, which is termed inconsistency factor (IF). Inconsistent loops were identified by a significant disagreement (IF and its 95% confidence interval that excludes 0) between direct and indirect evidence. *RAEs, renal adverse events.*

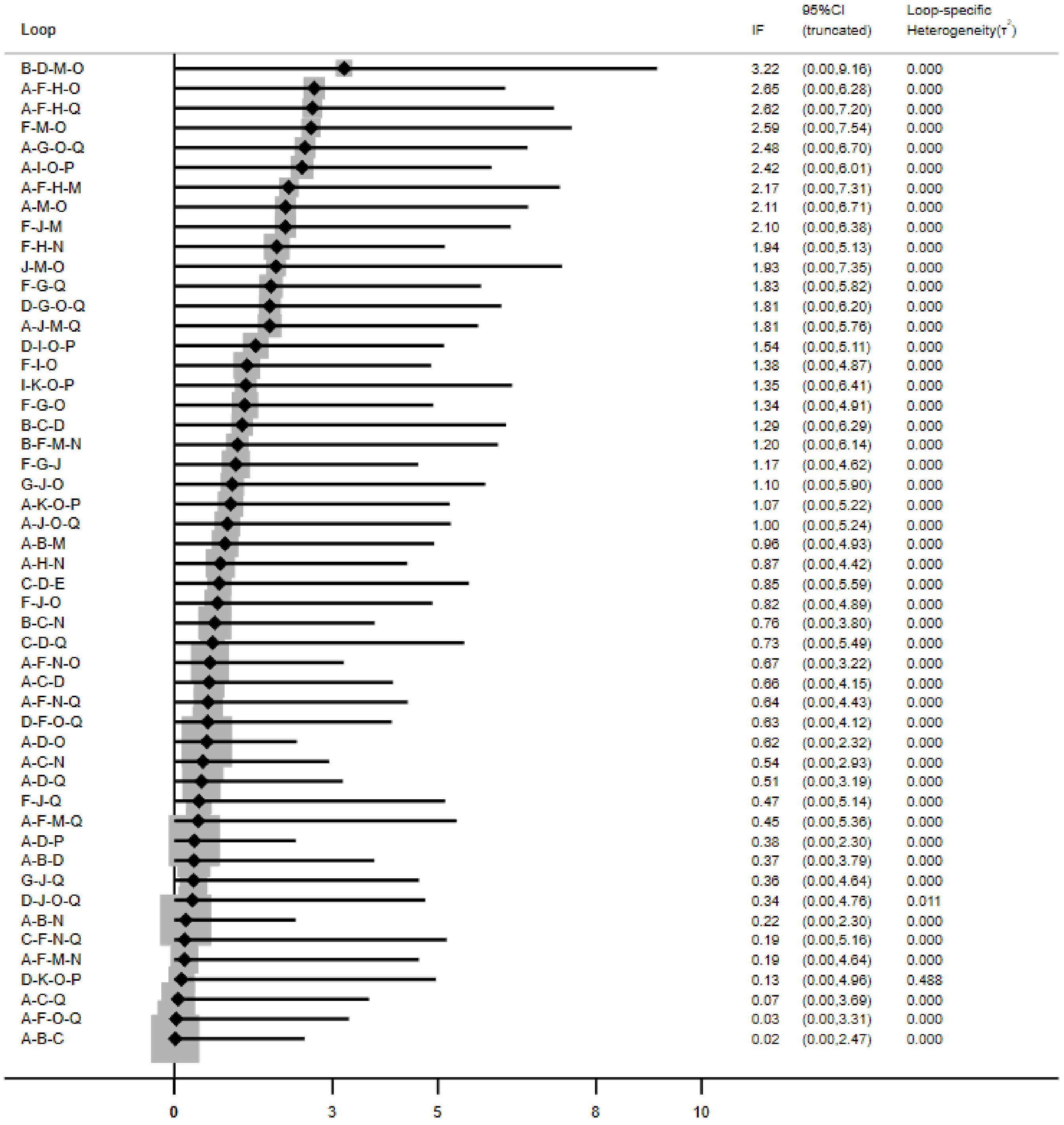

Supplemental Figure 10. The distribution of SUCRA values stratified by cancer types. *SUCRA, surface under the cumulative ranking; TTD, Targeted therapy drug; Chemo, Chemotherapy; PD-1, programmed cell death 1; PD-L1, programmed cell death ligand 1; CTLA4, cytotoxic T-lymphocyte antigen 4.*

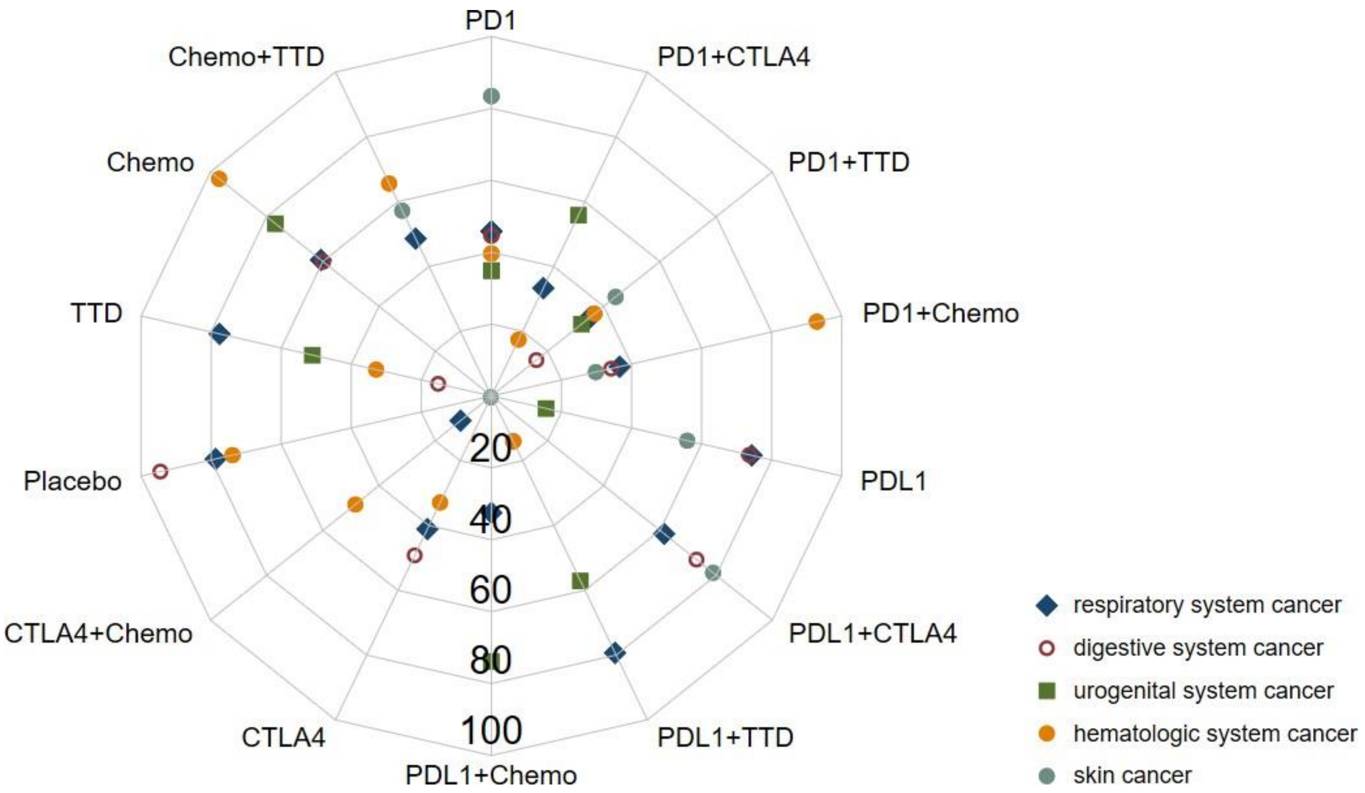

Table S1. PRISMA NMA Checklist of Items to Include When Reporting A Systematic Review Involving a Network Meta-analysis

Section/Topic	Item #	Checklist Item	Reported on page
**TITLE**			
Title	1	Identify the report as a systematic review *incorporating a network meta-analysis (or related form of meta-analysis).*	**1**
**ABSTRACT**			
Structured summary	2	Provide a structured summary including, as applicable: **Background:** main objectives<br><br>**Methods:** data sources; study eligibility criteria, participants, and interventions; study appraisal; and *synthesis methods, such as network meta-analysis.* **Results:** number of studies and participants identified; summary estimates with corresponding confidence/credible intervals; *treatment rankings may also be discussed. Authors may choose to summarize pairwise comparisons against a chosen treatment included in their analyses for brevity.* **Discussion/Conclusions:** limitations; conclusions and implications of findings. **Other:** primary source of funding; systematic review registration number with registry name.	**1**
**INTRODUCTION**			
Rationale	3	Describe the rationale for the review in the context of what is already known, *including mention of why a network meta-analysis has been conducted.*	**1-2**
Objectives	4	Provide an explicit statement of questions being addressed, with reference to participants, interventions, comparisons, outcomes, and study design (PICOS).	**2**
**METHODS**			
Protocol and registration	5	Indicate whether a review protocol exists and if and where it can be accessed (e.g., Web address); and, if available, provide registration information, including registration number.	**2**
Eligibility criteria	6	Specify study characteristics (e.g., PICOS, length of follow-up) and report characteristics (e.g., years considered, language, publication status) used as criteria for eligibility, giving rationale. *Clearly describe eligible treatments included in the treatment*	**3**

		network, and note whether any have been clustered or merged into the same node (with justification).	
Information sources	7	Describe all information sources (e.g., databases with dates of coverage, contact with study authors to identify additional studies) in the search and date last searched.	**3**
Search	8	Present full electronic search strategy for at least one database, including any limits used, such that it could be repeated.	**3, Table S2**
Study selection	9	State the process for selecting studies (i.e., screening, eligibility, included in systematic review, and, if applicable, included in the meta-analysis).	**3**
Data collection process	10	Describe method of data extraction from reports (e.g., piloted forms, independently, in duplicate) and any processes for obtaining and confirming data from investigators.	**3**
Data items	11	List and define all variables for which data were sought (e.g., PICOS, funding sources) and any assumptions and simplifications made.	**3**
**Geometry of the network**	**S1**	Describe methods used to explore the geometry of the treatment network under study and potential biases related to it. This should include how the evidence base has been graphically summarized for presentation, and what characteristics were compiled and used to describe the evidence base to readers.	**3-4**
Risk of bias within individual studies	12	Describe methods used for assessing risk of bias of individual studies (including specification of whether this was done at the study or outcome level), and how this information is to be used in any data synthesis.	**3-4**
Summary measures	13	State the principal summary measures (e.g., risk ratio, difference in means). *Also describe the use of additional summary measures assessed, such as treatment rankings and surface under the cumulative ranking curve (SUCRA) values, as well as modified approaches used to present summary findings from meta-analyses.*	**3-4**
Planned methods of analysis	14	Describe the methods of handling data and combining results of studies for each network meta-analysis. This should include, but not be limited to:<br>• *Handling of multi-arm trials;*<br>• *Selection of variance structure;*<br>• *Selection of prior distributions in Bayesian analyses; and*<br>• *Assessment of model fit.*	**3-4**
**Assessment of Inconsistency**	**S2**	Describe the statistical methods used to evaluate the agreement of direct and indirect evidence in the treatment network(s) studied. Describe efforts taken to address its presence when found.	**3-4**

Risk of bias across studies	15	Specify any assessment of risk of bias that may affect the cumulative evidence (e.g., publication bias, selective reporting within studies).	**3-4**
Additional analyses	16	Describe methods of additional analyses if done, indicating which were pre-specified. This may include, but not be limited to, the following:<br>• Sensitivity or subgroup analyses;<br>• Meta-regression analyses;<br>• *Alternative formulations of the treatment network; and*<br>• *Use of alternative prior distributions for Bayesian analyses (if applicable).*	**3-4**
**RESULTS†**			
Study selection	17	Give numbers of studies screened, assessed for eligibility, and included in the review, with reasons for exclusions at each stage, ideally with a flow diagram.	**4, Figure 1**
**Presentation of network structure**	**S3**	Provide a network graph of the included studies to enable visualization of the geometry of the treatment network.	**Figure 2, Supplemental Figure 4**
**Summary of network geometry**	**S4**	Provide a brief overview of characteristics of the treatment network. This may include commentary on the abundance of trials and randomized patients for the different interventions and pairwise comparisons in the network, gaps of evidence in the treatment network, and potential biases reflected by the network structure.	**4**
Study characteristics	18	For each study, present characteristics for which data were extracted (e.g., study size, PICOS, follow-up period) and provide the citations.	**4, Table S4**
Risk of bias within studies	19	Present data on risk of bias of each study and, if available, any outcome level assessment.	**4, Supplemental Figure 2, Supplemental Figure 3**
Results of individual studies	20	For all outcomes considered (benefits or harms), present, for each study: 1) simple summary data for each intervention group, and 2) effect estimates and confidence intervals. *Modified approaches may be needed to deal with information from larger networks.*	**4-5**
Synthesis of results	21	Present results of each meta-analysis done, including confidence/credible intervals. *In larger networks, authors may focus on comparisons versus a particular comparator (e.g. placebo or standard care), with full findings presented in an appendix. League tables and forest plots may be considered to summarize pairwise comparisons.* If additional summary measures were explored (such as treatment rankings), these should also be presented.	**4-6, Table 1, Table S5, Table S6**

**Exploration for inconsistency**	**S5**	Describe results from investigations of inconsistency. This may include such information as measures of model fit to compare consistency and inconsistency models, *P* values from statistical tests, or summary of inconsistency estimates from different parts of the treatment network.	**6, Supplemental Figure 9, Table S8**
Risk of bias across studies	22	Present results of any assessment of risk of bias across studies for the evidence base being studied.	**6, Supplemental Figure 8**
Results of additional analyses	23	Give results of additional analyses, if done (e.g., sensitivity or subgroup analyses, meta-regression analyses, *alternative network geometries studied, alternative choice of prior distributions for Bayesian analyses*, and so forth).	**6, Table S9, Table S10, Table S11**
**DISCUSSION**			
Summary of evidence	24	Summarize the main findings, including the strength of evidence for each main outcome; consider their relevance to key groups (e.g., healthcare providers, users, and policy-makers).	**6-8**
Limitations	25	Discuss limitations at study and outcome level (e.g., risk of bias), and at review level (e.g., incomplete retrieval of identified research, reporting bias). *Comment on the validity of the assumptions, such as transitivity and consistency. Comment on any concerns regarding network geometry (e.g., avoidance of certain comparisons).*	**8**
Conclusions	26	Provide a general interpretation of the results in the context of other evidence, and implications for future research.	**8**
**FUNDING**			
Funding	27	Describe sources of funding for the systematic review and other support (e.g., supply of data); role of funders for the systematic review. This should also include information regarding whether funding has been received from manufacturers of treatments in the network and/or whether some of the authors are content experts with professional conflicts of interest that could affect use of treatments in the network.	**8**

Table S2. Search strategy

A: Search strategy in PubMed

#	Query
1#	Programmed death ligand 1[tiab] OR PD-L1[tiab] OR Programmed death 1[tiab] OR PD-1[tiab] OR Cytotoxic T-lymphocyte antigen 4[tiab] OR CTLA-4[tiab] OR Nivolumab[tiab] OR Pembrolizumab[tiab] OR Cemiplimab[tiab] OR Toripalimab[tiab] OR Sintilimab[tiab] OR Atezolizumab[tiab] OR Avelumab[tiab] OR Durvalumab[tiab] OR Ipilimumab[tiab] OR Tremelimumab[tiab]
2#	randomized controlled trial[tiab] OR randomized[tiab] OR random[tiab] OR placebo[tiab] OR double blind[tiab]
3#	Humans[mh]
4#	1# and 2# and 3#
5#	2000/01/01[PDAT] : 2020/06/10[PDAT]
6#	4# and 5#

B: Search strategy in Embase

#	Query
1#	'programmed death ligand 1':ab,ti OR 'PD-L1':ab,ti OR 'programmed death 1':ab,ti OR 'PD-1':ab,ti OR ' Cytotoxic T-lymphocyte antigen 4 ' :ab,ti OR ' CTLA-4 ' :ab,ti OR ' Nivolumab ' :ab,ti OR 'Pembrolizumab':ab,ti OR 'Cemiplimab ab':ab,ti OR 'Toripalimab':ab,ti OR 'Sintilimab':ab,ti OR ' Atezolizumab ' :ab,ti OR ' Avelumab ' :ab,ti OR ' Durvalumab ' :ab,ti OR ' Ipilimumab ' :ab,ti OR 'Tremelimumab':ab,ti
2#	'randomized controlled trial':ab,ti OR 'randomized':ab,ti OR 'random':ab,ti OR 'placebo':ab,ti OR 'double blind':ab,ti
3#	2# and 3# AND [humans]/lim AND [1-1-2000]/sd NOT [11-6-2020]/sd

Ｃ: Search strategy in Cochrane Library

#	Query
1#	(programmed death ligand 1):ti,ab,kw OR(PD-L1):ti,ab,kw OR(programmed death 1):ti,ab,kw OR(PD-1):ti,ab,kw OR(Cytotoxic T-lymphocyte antigen 4):ti,ab,kw OR(CTLA-4):ti,ab,kw OR(Nivolumab):ti,ab,kw OR(Pembrolizumab):ti,ab,kw OR(Cemiplimab):ti,ab,kw OR(Toripalimab):ti,ab,kw OR(Sintilimab):ti,ab,kw OR(Atezolizumab):ti,ab,kw OR(Avelumab):ti,ab,kw OR(Durvalumab):ti,ab,kw OR(Ipilimumab):ti,ab,kw OR(Tremelimumab):ti,ab,kw
2#	(randomized controlled trial):ti,ab,kw OR(randomized):ti,ab,kw OR(random):ti,ab,kw OR(placebo):ti,ab,kw OR(double blind):ti,ab,kw
3#	2# and 3#
4#	Publication Date: Jan 2000 to Jun 2020

Table S3. FDA-approved doses of immune checkpoint inhibitors

CTLA-4 inhibitor	Ipilimumab	Administer 3 mg/kg as an intravenous infusion over 90 minutes every 3 weeks for a total of four doses.
PD-1 inhibitor	Nivolumab	Administer as an intravenous infusion over 30 minutes.<br>● 3 mg/kg every 2 weeks, 240 mg every 2 weeks or 480 mg every 4 weeks.<br>● Nivolumab with ipilimumab: Nivolumab 1 mg/kg, followed by ipilimumab on the same day, every 3 weeks for 4 doses, then Nivolumab 240 mg every 2 weeks or 480 mg every 4 weeks.<br>● Nivolumab with ipilimumab: Nivolumab 3 mg/kg followed by ipilimumab 1 mg/kg on the same day every 3 weeks for 4 doses, then Nivolumab 240 mg every 2 weeks or 480 mg every 4 weeks.
	Pembrolizumab	Administer as an intravenous infusion over 30 minutes.<br>● 2 mg/kg every 3 weeks, 200 mg every 3 weeks or 400 mg every 6 weeks.
PD-L1 inhibitor	Avelumab	Administer as an intravenous infusion over 60 minutes.<br>● 10 mg/kg every2 weeks or 800 mg every 2 weeks.
	Atezolizumab	Administer as an intravenous infusion over 60 minutes. If the first infusion is tolerated, all subsequent infusions may be delivered over 30 minutes.<br>● 840 mg every 2 weeks, 1200 mg every 3 weeks, or 1680 mg every 4 weeks.
	Durvalumab	Administer 10 mg/kg as an intravenous infusion over 60 minutes every 2 weeks.

Table S4. Main characteristics of the trials included in this meta-analysis

Trial	Year	Multi center	Phase	Tumor type	Drug	Median age	Male(%)	Sample size	Number of patients	RAAEs		Acute kidney injury		CTCAE version
										All grade	Grade 3-5	All grade	Grade 3-5	
NCT02538666	2015	Y	3	SCLC	Placebo	63.2	63.6	830	273	7	1	0	0	4
					Niv	64.6	63.2		279	12	1	0	0	
					Niv+Ipi	63.9	64.5		278	23	5	2	2	
NCT01668784	2012	Y	3	RCC	Niv	60.6	76.8	803	406	73	18	8	8	4
					TTD	61.9	74		397	58	9	5	5	
Wolchok	2017	Y	3	Melanoma	Niv	60	64	937	313	2	1	-	-	4
					Niv+Ipi	61	66		313	14	1	-	-	
					Ipi	62	64		311	5	0	-	-	
NCT01621490	2012	Y	1	Melanoma	Niv	54.7	56.6	168	106	5	2	1	1	4
					Niv+Ipi	57.9	64.5		62	8	1	4	1	
Wu	2019	Y	3	NSCLC	Niv	60	78	493	337	16	0	1	0	4
					Chemo	60	81		156	5	0	0	0	
Borghaei	2015	Y	3	NSCLC	Niv	61	52	555	287	7	0	-	-	4
					Chemo	64	58		268	1	0	-	-	
NCT02481830	2015	Y	3	SCLC	Niv	61.5	61.3	547	282	1	1	-	-	4
					Chemo	61.6	62.1		265	1	1	-	-	
Ferris	2016	Y	3	HNSCC	Niv	59	82.1	347	236	1	0	1	0	4
					Chemo	61	85.1		111	2	1	2	1	
Brahmer	2015	Y	3	NSCLC	Niv	62	82	260	131	5	1	-	-	4
					Chemo	64	71		129	3	0	-	-	
Carbone	2017	Y	3	NSCLC	Niv	63	68	530	267	5	1	-	-	4
					Chemo	65	55		263	16	0	-	-	
Robert	2015	Y	3	Melanoma	Niv+Placebo	64	57.6	411	206	4	1	-	-	4
					Chemo+Placebo	66	60.1		205	1	0	-	-	
Weber	2015	Y	3	Melanoma	Niv	59	65	370	268	2	0	-	-	4
					Chemo	62	64		102	0	0	-	-	
NCT03361865	2017	Y	3	UC	Pem+TTD	73.3	75	92	43	5	2	0	0	4
					Pem+Placebo	72.4	77.6		49	7	4	2	2	
NCT03374488	2017	Y	3	UC	Pem+TTD	67.9	83.3	83	42	3	0	0	0	4

					Pem+Placebo	65.2	88.1		41	2	2	1	1	
NCT03322540	2017	Y	2	NSCLC	Pem+TTD	63.7	68.8	152	75	3	0	0	0	4
					Pem+ placebo	66.9	76.6		77	7	1	1	1	
Eggermont	2018	Y	3	Melanoma	Pem	54	63	1011	509	2	2	-	-	4
					Placebo	54	60.2		502	1	0	-	-	
NCT02337491	2015	Y	2	Glioblastoma	Pem+TTD	50.7	70	80	50	4	-	-	-	4
					Pem	51.8	63.3		30	0	-	-	-	
Long	2019	Y	3	Melanoma	Pem+TTD	64	61	705	353	5	2	1	1	4.03
					Pem+Placebo	63	59		352	5	1	3	1	
Robert	2015	Y	3	Melanoma	Ipi	62	58.3	533	256	2	1	-	-	4
					Pem	63	62.8		277	4	0	-	-	
NCT03358472	2017	Y	3	HNSCC	Pem+TTD	62.1	85.7	87	34	4	1	1	1	4
					Pem	63	84.2		19	1	0	0	0	
					Chemo	62.7	82.9		34	4	1	0	0	
NCT02702401	2016	Y	3	HCC	Pem	65.6	81.3	413	279	5	5	3	3	4
					Placebo	64.4	83		134	0	0	0	0	
Herbst	2016	Y	2/3	NSCLC	Pem	63	62	648	339	6	0	-	-	4
					Chemo	62	61		309	0	0	-	-	
NCT02546986	2015	Y	2	NSCLC	Pem+Chemo	62.8	51	100	51	0	0	0	0	4
					Pem+Placebo	64.9	63.3		49	2	2	1	1	
NCT02351739	2015	Y	2	UC	Pem	65.8	20	75	35	9	2	4	2	4.03
					Pem+TTD	66.4	27.5		40	12	5	7	5	
Bellmunt	2017	Y	3	UC	Pem	67	74.1	521	266	30	11	15	7	4
					Chemo	65	74.3		255	22	4	7	3	
NCT02448303	2015	N	2	NSCLC	Pem	63.7	54.5	68	33	5	1	3	1	4.03
					Pem+TTD	64.6	42.9		35	8	0	4	0	
Burtness	2019	Y	3	HNSCC	Pem	62	83	863	300	9	6	4	4	4
					Pem+Chemo	61	80		276	38	6	5	5	
					Chemo+TTD	61	87		287	19	2	2	2	
NCT02494583	2015	Y	3	GEC	Pem	59.9	70.3	748	254	16	8	4	4	4
					Pem+Chemo	60.9	75.9		250	43	13	8	8	
					Chemo+Placebo	60.7	71.6		244	48	14	8	8	

NCT02454179	2015	N	2	HNSCC	Pem	62.1	87.2	76	39	2	0	0	0	4.03
					Pem+TTD	61.4	94.6		37	12	3	6	3	
Mok	2019	Y	3	NSCLC	Pem	63	71	1251	636	3	1	-	-	4
					Chemo	63	71		615	0	0	-	-	
Cohen	2019	Y	3	HNSCC	Pem	60	84	480	246	1	1	1	1	4
					Chemo	60	83		234	3	0	1	0	
NCT03933449	2016	Y	3	GEC	Pem	60.1	91.9	121	62	1	1	1	1	4
					Chemo	59.6	95.1		59	0	0	0	0	
NCT03850444	2016	Y	3	NSCLC	Pem	60.9	82	253	128	2	2	-	-	4
					Chemo	61.5	88.8		125	1	1	-	-	
NCT02564263	2015	Y	3	GEC	Pem	62.6	86.9	610	314	2	2	2	2	4
					Chemo	62	86.3		296	2	2	1	1	
Ribas	2015	Y	2	Melanoma	Pem	62	58	349	178	1	0	-	-	4
					Chemo	63	64		171	0	0	-	-	
NCT02370498	2015	Y	3	GEC	Pem	60.7	68.2	570	294	5	5	3	3	4
					Chemo	59.6	70.3		276	2	2	0	0	
Reck	2016	Y	3	NSCLC	Pem	64.5	59.7	304	154	4	1	-	-	3
					Chemo	66	62.9		150	15	1	-	-	
NCT01454102	2011	Y	1	NSCLC	Niv+Chemo	61.4	47.6	203	56	10	3	2	1	4
					Niv+TTD	62.8	45.5		33	4	2	0	0	
					Niv	66.2	51.1		90	3	0	0	0	
					Niv+Ipi	60.5	58.3		24	3	1	0	0	
NCT03023423	2016	Y	1b/2	NSCLC	Ate	61.7	60.9	88	44	1	1	1	1	-
					Ate+TTD	63.2	82.6		44	1	0	0	0	
NCT02008227	2013	Y	3	NSCLC	Chemo	62.9	61.9	1187	578	4	4	4	4	4
					Ate	62.7	61.8		609	3	3	1	1	
NCT02302807	2015	Y	3	UC	Chemo	66.1	77.8	902	443	23	12	6	6	4
					Ate	65.9	76.4		459	54	18	11	11	
NCT01984242	2014	Y	2	RCC	Ate+TTD	61.1	73.3	304	101	14	1	1	1	-
					Ate	60.1	74.8		103	21	4	3	3	
					TTD	59.7	78.2		100	18	4	1	1	
NCT01903993	2013	Y	2	NSCLC	Chemo	61.8	53.1	277	135	2	2	1	1	4

					Ate	61.5	64.6		142	1	1	1	1	
Eng	2019	Y	3	CRC	TTD	59	57	349	80	0	0	0	0	4
					Ate+TTD	58	58		179	2	2	1	1	
					Ate	56	66		90	0	0	0	0	
NCT02625623	2015	Y	3	GEC	Chemo	60.1	68.3	361	177	2	2	1	1	4.03
					Ave	58.8	75.7		184	0	0	0	0	
Barlesi	2018	Y	3	NSCLC	Ave	64	68	758	393	1	1	1	1	4.03
					Chemo	63	69		365	2	1	0	0	
NCT02580058	2015	Y	3	OC	Ave	61	0	546	187	2	2	1	1	4.03
					Ave+Chemo	59.5	0		182	1	1	0	0	
					Chemo	60.4	0		177	1	1	0	0	
NCT02336165	2015	Y	2	Glioblastoma	Dur	54	83.9	119	31	1	1	-	-	-
					Dur+TTD	55.8	59.1		88	0	0	-	-	
NCT02558894	2015	Y	2	PC	Dur+Tre	61.3	46.9	64	32	0	0	0	0	4.03
					Dur	61.6	57.6		32	2	2	2	2	
Antonia	2017	Y	3	NSCLC	Dur	64	70.2	709	475	5	5	2	2	4.03
					Placebo	64	70		234	0	0	0	0	
NCT02369874	2015	Y	3	HNSCC	Dur+Tre	59.9	84.6	723	246	1	1	1	1	4.03
					Dur	59	84.2		237	2	2	0	0	
					Chemo	59.5	83.1		240	1	1	0	0	
NCT02453282	2015	Y	3	NSCLC	Dur	63.2	68.4	1092	369	12	1	5	1	4.03
					Dur+Tre	64.3	71.5		371	30	2	5	0	
					Chemo	63.6	67.2		352	40	3	5	1	
NCT02319044	2015	Y	2	HNSCC	Dur+Tre	62	85	263	133	0	0	-	-	4.03
					Dur	62	80.6		65	0	0	-	-	
					Tre	61	79.1		65	1	1	-	-	
NCT02352948	2015	Y	3	NSCLC	Dur	63.2	64.2	585	179	6	0	0	0	-
					Chemo	63.9	70.9		173	8	1	0	0	
					Dur+Tre	62.5	66.1		173	5	3	1	1	
					Tre	63.5	65		60	2	0	0	0	
NCT02409355	2015	Y	3	NSCLC	Ate	-	50	8	4	3	1	-	-	4
					Chemo	-	100		4	0	0	-	-	

NCT00094653	2004	Y	2	Melanoma	Ipi	56.8	59.1	643	131	5	5	-	-	3
					Ipi+TTD	55.6	61.3		380	4	4	-	-	
					TTD	57.4	53.7		132	1	1	-	-	
NCT01740297	2013	Y	1b/2	Melanoma	Ipi	64.2	55	190	95	0	0	0	-	-
					Ipi+TTD	63.6	63.3		95	2	1	1	-	
Le	2013	N	1	PC	Ipi	63	73	30	15	1	1	-	-	3
					Ipi+TTD	62	67		15	0	0	-	-	
Hodi	2014	Y	2	Melanoma	Ipi+TTD	61	69.1	238	118	1	1	0	0	4
					Ipi	64	63.9		120	1	1	1	1	
NCT01471197	2012	Y	2	NSCLC	Ipi	62.7	66.7	8	6	1	-	-	-	-
					Chemo	61	50		2	0	-	-	-	
NCT01585987	2012	Y	2	GEC	Ipi	65	63.2	108	57	1	1	1	1	-
					Chemo	62	71.9		45	0	0	0	0	
					Placebo	-	-		6	0	0	0	0	
NCT01708941	2013	Y	2	Melanoma	Ipi+TTD	65	68.4	43	21	1	0	0	0	4
					Ipi	57	59.1		22	2	1	1	1	
NCT00636168	2008	Y	3	Melanoma	Ipi	50.7	62.3	945	471	3	3	-	-	3
					Placebo	51.5	61.6		474	0	0	-	-	
NCT02158520	2013	Y	2	Melanoma	Chemo	60	66.7	24	12	1	0	0	0	4
					Ipi	61	58.3		12	3	3	3	3	
NCT01057810	2010	Y	3	PCa	Placebo	68.6	100	598	199	2	2	-	-	3
					Ipi	69.3	100		399	16	16	-	-	
NCT00796991	2009	Y	1	Melanoma	Ipi+Chemo	54.5	66.7	59	39	4	1	-	-	
					Ipi	60	60		20	3	1	-	-	
Maio	2017	Y	2	MESO	Placebo	67	80	569	189	4	2	2	1	3
					Tre	66	74		380	20	1	12	5	
Postow	2015	Y	2	Melanoma	Niv+Ipi	64	66.3	140	94	3	1	-	-	4
					Ipi	67	68.1		46	1	0	-	-	
Motzer	2019	Y	3	RCC	Niv+Ipi	61.1	75.1	1082	547	52	5	12	4	4
					TTD	60.7	72.3		535	45	5	9	3	
NCT01472081	2012	Y	1	RCC	Niv+TTD	57.4	83	100	53	22	4	7	3	4
					Niv+Ipi	55.6	80.9		47	12	1	3	1	

Langer	2016	Y	1/2	NSCLC	Pem+Chemo	62.5	37	121	59	8	2	2	2	4
					Chemo	63.2	41		62	5	0	1	0	
NCT01928394	2013	Y	1/2	Solid Tumor	Niv	61.8	62	793	418	32	11	17	8	4
					Niv+Ipi	61	57.1		375	29	9	13	5	
NCT02011945	2014	Y	1	CML	TTD	48.5	50	18	2	0	-	-	-	4.03
					Niv+TTD	47.1	68.8		16	2	-	-	-	
NCT02302339	2014	Y	2	Melanoma	TTD	-	55.3	132	103	12	1	6	0	4
					Niv+TTD	-	62.1		29	4	2	3	2	
NCT03260894	2017	Y	3	RCC	Pem+TTD	62.9	68.8	127	64	7	-	-	-	4
					TTD	62.1	76.9		63	7	-	-	-	
Usmani	2019	Y	3	MM	Pem+Chemo	74	46	294	149	6	6	6	6	4
					Chemo	74	47		145	1	1	1	1	
Mateos	2019	Y	3	MM	Pem+Chemo	65	62	241	120	4	4	4	4	4
					Chemo	67	63		121	4	4	4	4	
Rini	2019	Y	3	RCC	Pem+TTD	62	71.3	854	429	54	3	-	-	4
					TTD	61	74.6		425	52	3	-	-	
Paz-Ares	2018	Y	3	NSCLC	Pem+Chemo	65	79.1	558	278	2	2	-	-	4.03
					Chemo	65	83.6		280	2	2	-	-	
NCT02537444	2015	N	2	OC	TTD	64.6	-	77	38	2	0	0	0	4.03
					Pem+TTD	64.2	-		39	4	1	1	1	
NCT02362048	2015	N	2	PC	TTD	62.9	48.6	73	35	7	0	2	0	4.03
					Pem+TTD	61.3	50		38	8	1	4	1	
Horn	2018	Y	3	SCLC	Ate+Chemo	64	64.2	394	198	7	4	4	2	4
					Chemo+Placebo	64	65.3		196	2	0	1	0	
West	2019	Y	3	NSCLC	Ate+Chemo	64	57	705	473	54	13	9	4	4
					Chemo	65	58		232	18	2	3	1	
NCT02367794	2015	Y	3	NSCLC	Chemo	64.9	81.5	1000	334	4	1	1	1	4
					Ate+Chemo	64.5	81.8		666	43	7	6	6	
NCT02420821	2015	Y	3	RCC	TTD	59.9	76.4	897	446	49	13	1	1	4
					Ate+TTD	61.6	69.8		451	51	16	9	9	
NCT02454933	2015	Y	3	NSCLC	TTD	62.3	23.5	29	17	0	-	-	-	4.03
					Dur+TTD	57.6	50		12	1	-	-	-	

NCT02250326	2015	Y	2	NSCLC	Chemo	63.3	62.1	236	158	3	2	2	2	4
					Dur+Chemo	62.7	68.4		78	4	0	0	0	
NCT00324155	2006	Y	3	Melanoma	Ipi+Chemo	57.5	60.8	498	247	5	5	-	-	3
					Chemo+Placebo	56.4	59.1		251	4	4	-	-	
NCT02279732	2014	Y	3	NSCLC	Ipi+Chemo	60.9	88.8	204	98	2	2	1	1	3
					Chemo+Placebo	59.8	87.7		106	0	0	0	0	
Gandhi	2018	Y	3	NSCLC	Pem+Chemo	65	62	607	405	43	8	-	-	4
					Chemo+Placebo	63.5	52.9		202	11	0	-	-	
NCT02718417	2016	Y	3	OC	Ave+Chemo	58.3	-	662	328	1	1	1	1	4.03
					Chemo	57.1	-		334	0	0	0	0	
NCT01450761	2011	Y	3	SCLC	Ipi+Chemo	61.3	66.3	1123	562	9	9	4	4	3
					Chemo	62.6	68.5		561	3	3	1	1	
NCT03273153	2017	Y	3	Melanoma	Pem	63.5	62.9	436	216	1	1	0	0	4
					Ate+TTD	63.6	58.1		220	7	7	2	2	
NCT02555657	2015	Y	3	BC	Pem	51.4	-	601	309	1	1	0	0	4
					Chemo	52.6	0.6		292	2	2	2	2	
NCT02340975	2015	Y	1b/2	GEC	Dur	59.8	79.2	113	24	1	0	0	0	4.03
					Tre	52.3	66.7		12	3	3	1	1	
					Dur+Tre	60.4	72.7		77	8	3	4	2	

RAEs, renal adverse events; CTCAE, Common Terminology Criteria for Adverse Events; SCLC, small cell lung cancer; RCC, renal cell carcinoma; HNSCC, Head and Neck Squamous Cell Carcinoma; NSCLC, non-small cell lung cancer; UC, urothelial carcinoma; HCC, hepatocellular carcinoma; GEC, gastric or gastroesophageal junction cancer; CRC, Colorectal Cancer; OC, ovarian cancer; PC, pancreatic cancer; PCa, prostate cancer; MESO, mesothelioma; CML, chronic myeloid leukemia; MM, multiple myeloma; BC, breast cancer; Niv, Nivolumab; Ipi, Ipilimumab; TTD, Targeted therapy drug; Chemo, Chemotherapy; Pem, Pembrolizumab; Ate, Atezolizumab; Ave, Avelumab; Dur, Durvalumab; Tre, Tremelimumab.

Table S5. Results of traditional pairwise meta-analysis for RAEs

Comparisons	N	n	Tau[2a]	I^2	Q	RR (95%CI)[b]
PD1 vs. Placebo	3	1976	0	0	0.58	1.85 (0.81-4.20)
PD1+CTLA4 vs Placebo	1	551	NA	NA	NA	**3.06 (1.33-7.01)**
PDL1 vs. Placebo	1	709	NA	NA	NA	5.37 (0.30-96.8)
CTLA4 vs. Placebo	5	2438	0	0	2.63	**2.85 (1.35-6.01)**
PD1 vs. Chemo+TTD	4	1467	0	0	0.36	**0.43 (0.22-0.84)**
PD1+TTD vs. Chemo+TTD	1	68	NA	NA	NA	1.00 (0.27-3.71)
PD1+ Chemo vs. Chemo+TTD	1	563	NA	NA	NA	**1.95 (1.15-3.30)**
PDL1 vs. Chemo+TTD	2	829	0	0	0.58	0.86 (0.33-2.23)
CTLA4 vs. Chemo+TTD	2	257	0	0	0.90	1.12 (0.32-3.87)
CTLA4+ Chemo vs. Chemo+TTD	2	832	0	0	0.08	0.67 (0.24-1.87)
PD1 vs. Chemo	16	7893	0.28	33.9	22.70	1.16 (0.70-1.91)
PD1+ Chemo vs. Chemo	3	1093	0.04	4.1	2.09	1.49 (0.54-4.09)
PDL1 vs. Chemo	8	4578	1.04	73.9	26.83	0.81 (0.31-2.11)
PDL1+ Chemo vs. Chemo	5	2883	0.16	29.0	5.64	**1.99 (1.03-3.85)**
PDL1+ CTLA4 vs. Chemo	1	723	NA	NA	NA	0.73 (0.47-1.15)
CTLA4 vs. Chemo	2	110	0	0	0.11	1.57 (0.18-13.5)
CTLA4+ Chemo vs. Chemo	1	1123	NA	NA	NA	2.96 (0.81-10.9)
PD1 vs. TTD	1	803	NA	NA	NA	1.20 (0.87-1.65)
PD1+CTLA4 vs. TTD	1	1082	NA	NA	NA	1.12 (0.76-1.64)
PD1+TTD vs. TTD	6	1281	0	0	0.57	1.05 (0.78-1.41)
PDL1 vs. TTD	2	373	NA	NA	NA	1.11 (0.62-1.98)
PDL1+TTD vs. TTD	4	1386	0	0	1.47	0.99 (0.72-1.36)
CTLA4 vs. CTLA4+ Chemo	1	59	NA	NA	NA	1.40 (0.34-5.74)
PD1 vs. CTLA4	2	1157	0.44	37.9	1.61	0.84 (0.19-3.75)
PD1+ CTLA4 vs. CTLA4	1	624	NA	NA	NA	2.71 (0.99-7.42)
PDL1 vs. CTLA4	3	405	0	0	1.49	0.54 (0.16-1.75)
PDL1+CTLA4 vs. CTLA4	3	520	0	0	0.91	0.53 (0.21-1.33)
PDL1 vs. PDL1+ Chemo	1	369	NA	NA	NA	1.94 (0.18-21.2)
PD1 vs. PDL1+ TTD	1	436	NA	NA	NA	0.15 (0.02-1.20)
PDL1 vs. PDL1+ TTD	4	680	0	0	1.89	1.39 (0.77-2.50)
PDL1 vs. PDL1+ CTLA4	6	1938	0.18	24.3	5.29	0.72 (0.35-1.49)
PD1 vs. PD1+ Chemo	3	1226	0	0	1.72	**0.32 (0.21-0.48)**
PD1+CTLA4 vs. PD1+ Chemo	1	80	NA	NA	NA	0.73 (0.33-2.46)
PD1+TTD vs. PD1+ Chemo	1	89	NA	NA	NA	0.71 (0.24-2.12)
PD1 vs. PD1+ TTD	6	475	0	0	5.00	**0.57 (0.34-0.94)**
PD1+CTLA4 vs. PD1+ TTD	2	157	0	0	0.25	0.74 (0.42-1.30)
PD1 vs. PD1+CTLA4	5	2258	0.25	55.7	9.03	**0.49 (0.27-0.91)**
Chemo vs. Placebo	1	51	NA	NA	NA	NA

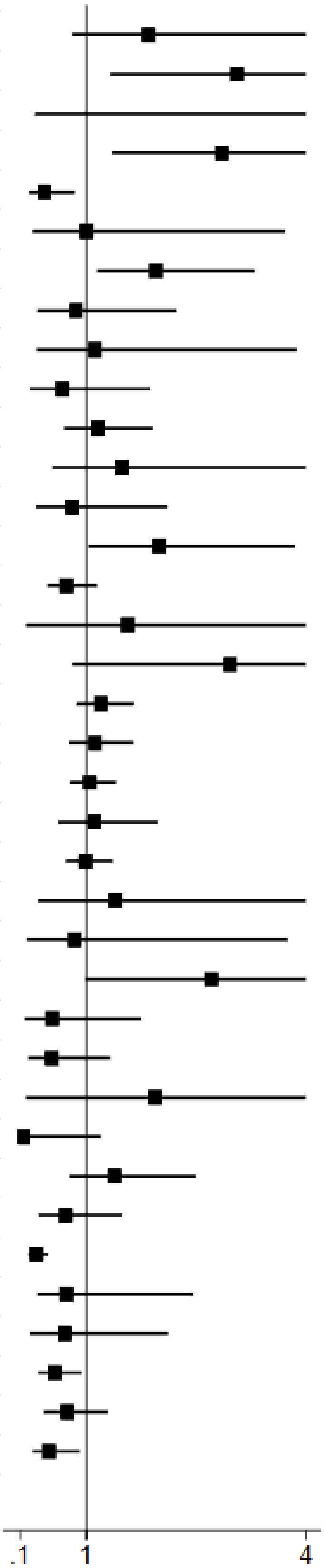

RAEs, renal adverse events; N, number of trials; n, number of patients; RR, risk ratio; CI, confidence interval; PD-1, programmed cell death 1; PD-L1, programmed cell death ligand 1; CTLA4, cytotoxic T-lymphocyte antigen 4; Chemo, Chemotherapy; TTD, Targeted therapy drug; NA, no available.

[a] Tau^2 represents between-study heterogeneity characterized by standard deviation.

[b] Bold values denote statistical significance.

Table S6. Network estimates of treatment comparisons for AKI and grade 3-5 AKI

**PD-1**	1.40 (0.57-3.60)	0.67 (0.27-1.65)	**0.44 (0.18-0.95)**	0.81 (0.32-2.07)	1.61 (0.37-8.25)	1.23 (0.39-4.38)	0.76 (0.24-2.47)	0.41 (0.09-1.76)	0.34 (0.05-1.93)	**4.40 (1.78-13.0)**	0.63 (0.29-1.25)	0.65 (0.23-1.79)	**9.71 (1.43-321)**
1.06 (0.52-2.04)	**PD1+ CTLA4**	0.48 (0.15-1.48)	**0.31 (0.09-0.98)**	0.57 (0.17-2.03)	1.15 (0.20-7.12)	0.88 (0.22-3.82)	0.54 (0.12-2.28)	0.29 (0.05-1.56)	0.24 (0.03-1.64)	**3.12 (1.07-10.8)**	0.45 (0.13-1.32)	0.46 (0.11-1.69)	6.96 (0.84-233)
0.73 (0.38-1.37)	0.69 (0.31-1.51)	**PD1+ TTD**	0.65 (0.19-2.01)	1.20 (0.35-4.09)	2.41 (0.44-14.9)	1.84 (0.46-7.91)	1.14 (0.26-4.85)	0.61 (0.11-3.22)	0.50 (0.06-3.46)	**6.58 (2.15-23.1)**	0.93 (0.29-2.75)	0.96 (0.26-3.39)	**14.5 (1.74-489)**
**0.45 (0.20-0.93)**	0.43 (0.15-1.12)	0.62 (0.23-1.55)	**PD1+ Chemo**	1.86 (0.67-5.66)	3.72 (0.79-20.7)	2.83 (0.77-12.9)	1.75 (0.52-6.40)	0.93 (0.20-4.62)	0.77 (0.11-4.90)	**10.1 (3.21-41.8)**	1.44 (0.62-3.40)	1.48 (0.49-4.69)	**22.4 (2.87-841)**
0.77 (0.33-1.67)	0.73 (0.27-1.99)	1.05 (0.39-2.81)	1.68 (0.67-4.40)	**PDL1**	1.99 (0.51-8.84)	1.54 (0.48-5.24)	0.94 (0.27-3.17)	0.50 (0.12-2.12)	0.41 (0.06-2.50)	**5.40 (1.81-19.3)**	0.78 (0.34-1.59)	0.80 (0.24-2.56)	**12.0 (1.63-414)**
0.84 (0.27-2.44)	0.79 (0.23-2.81)	1.15 (0.33-3.96)	1.84 (0.57-6.23)	1.09 (0.43-2.82)	**PDL1+ CTLA4**	0.76 (0.13-4.58)	0.47 (0.08-2.63)	0.25 (0.05-1.34)	0.21 (0.02-1.77)	2.73 (0.46-16.2)	0.39 (0.08-1.57)	0.40 (0.07-1.88)	6.14 (0.58-245)
0.90 (0.29-2.64)	0.85 (0.25-2.92)	1.24 (0.36-4.05)	1.97 (0.55-7.30)	1.17 (0.38-3.68)	1.07 (0.26-4.43)	**PDL1+ TTD**	0.61 (0.12-2.89)	0.32 (0.06-1.89)	0.27 (0.03-2.03)	**3.52 (1.13-12.6)**	0.50 (0.13-1.66)	0.52 (0.11-2.22)	7.98 (0.84-298)
0.72 (0.26-1.87)	0.67 (0.20-2.14)	0.98 (0.30-3.06)	1.57 (0.56-4.63)	0.93 (0.33-2.59)	0.84 (0.24-3.11)	0.81 (0.20-3.11)	**PDL1+ Chemo**	0.54 (0.09-3.02)	0.44 (0.06-2.89)	**5.80 (1.44-27.4)**	0.82 (0.30-2.17)	0.84 (0.20-3.58)	**12.9 (1.41-493)**
0.46 (0.12-1.71)	0.43 (0.10-1.87)	0.62 (0.15-2.67)	1.00 (0.26-4.28)	0.59 (0.17-2.23)	0.54 (0.14-2.29)	0.51 (0.10-2.67)	0.63 (0.14-3.03)	**CTLA4**	0.83 (0.08-7.12)	**10.7 (2.10-63.3)**	1.53 (0.35-6.48)	1.57 (0.35-6.76)	**24.4 (2.89-894)**
0.38 (0.06-2.11)	0.36 (0.05-2.18)	0.52 (0.08-3.21)	0.84 (0.13-5.01)	0.49 (0.08-2.72)	0.46 (0.06-2.86)	0.42 (0.05-2.91)	0.53 (0.08-3.28)	0.82 (0.09-6.43)	**CTLA4+ Chemo**	**13.3 (1.92-119)**	1.88 (0.34-11.1)	1.92 (0.27-15.2)	**30.7 (2.27-1560)**
**2.18 (1.07-4.67)**	2.07 (0.97-4.89)	**3.01 (1.44-6.68)**	**4.82 (1.83-14.5)**	**2.85 (1.08-7.98)**	2.61 (0.77-9.58)	2.45 (0.86-7.48)	3.06 (0.98-10.6)	**4.80 (1.12-20.7)**	5.86 (0.92-41.4)	**TTD**	**0.14 (0.04-0.40)**	**0.15 (0.03-0.53)**	2.20 (0.25-74.6)
0.72 (0.36-1.29)	0.68 (0.26-1.59)	0.98 (0.39-2.25)	1.57 (0.74-3.38)	0.93 (0.47-1.76)	0.85 (0.31-2.26)	0.80 (0.24-2.47)	1.00 (0.42-2.27)	1.59 (0.40-5.37)	1.87 (0.38-10.6)	**0.33 (0.12-0.77)**	**Chemo**	1.03 (0.35-3.19)	**15.6 (2.17-561)**
0.59 (0.23-1.48)	0.57 (0.18-1.72)	0.82 (0.27-2.39)	1.31 (0.48-3.78)	0.77 (0.26-2.27)	0.71 (0.20-2.48)	0.66 (0.17-2.71)	0.83 (0.23-3.00)	1.30 (0.33-4.89)	1.58 (0.24-11.3)	**0.27 (0.08-0.84)**	0.83 (0.31-2.32)	**Chemo+ TTD**	**15.5 (1.88-530)**
**4.58 (1.00-38.3)**	4.37 (0.86-39.6)	**6.34 (1.24-57.8)**	**10.2 (1.98-96.8)**	**6.03 (1.21-52.6)**	**5.58 (1.01-52.8)**	5.27 (0.86-52.3)	**6.45 (1.13-65.4)**	**10.1 (2.21-80.1)**	**12.8 (1.16-201)**	2.10 (0.41-19.0)	**6.43 (1.39-58.2)**	**7.80 (1.43-76.4)**	**Placebo**

Network estimates of treatment comparisons for AKI (on the lower triangle) and grade 3-5 AKI (on the upper triangle). The summary estimates are risk ratios (RRs) and 95% confidence intervals. For AKI, the column-defining treatment is compared to the row-defining treatment, and RRs < 1 favor the column-defining treatment. For grade 3-5 AKI, the row-defining treatment is compared to the column-defining treatment, and RRs < 1 favor the row-defining treatment.

Significant results are in bold.

AKI, acute kidney injury; PD-1, programmed cell death 1; PD-L1, programmed cell death ligand 1; CTLA4, cytotoxic T-lymphocyte antigen 4; Chemo, Chemotherapy; TTD, Targeted therapy drug.

Table S7. Evaluation of the model fit

Model assumption	Dbar	Pd	# of data points	Deviance Information Criterion
Random consistency	232	122	208	354
Random inconsistency	226	136	208	362
Fixed consistency	306	97	208	403
Fixed inconsistency	284	118	208	402

Deviance Information Criterion (DIC) is the sum of the posterior mean of the residual deviance and the Pd, and provides a measure of model fit that penalizes model complexity - lower values of the DIC suggest a more parsimonious model. The DIC is particularly useful for comparing different parameter models for the same likelihood and data. As shown in this table, the random consistency model is more parsimonious than the other three models.

Table S8. Evaluation of local inconsistency for RAEs using node-split model

Comparison	Direct risk ratio	Indirect risk ratio	Ratio of risk ratios	P-value
CTLA4 vs Chemo	0.65 (0.07-6.17)	1.58 (0.84-3.01)	0.41 (0.04-4.22)	0.426
CTLA4+Chemo vs Chemo	2.40 (0.54-12.3)	1.25 (0.39-3.97)	1.92 (0.27-13.4)	0.494
PD1 vs Chemo	0.92 (0.60-1.41)	0.46 (0.24-0.87)	2.00 (0.92-4.33)	0.073
PD1+Chemo vs Chemo	1.20 (0.44-3.34)	2.43 (1.29-4.51)	0.49 (0.15-1.63)	0.246
PDL1 vs Chemo	0.75 (0.42-1.29)	1.39 (0.63-3.14)	0.54 (0.20-1.44)	0.205
PDL1+Chemo vs Chemo	1.73 (0.88-3.36)	4.05 (0.70-27.7)	0.43 (0.06-3.02)	0.376
PDL1+CTLA4 vs Chemo	0.69 (0.28-1.69)	0.73 (0.31-1.67)	0.95 (0.28-3.24)	0.927
CTLA4 vs Chemo+TTD	0.95 (0.25-3.22)	0.90 (0.36-2.27)	1.06 (0.22-5.10)	0.952
PD1 vs Chemo+TTD	0.32 (0.13-0.76)	0.59 (0.22-1.63)	0.54 (0.14-2.06)	0.363
PD1+Chemo vs Chemo+TTD	2.00 (0.70-5.80)	0.97 (0.39-2.35)	2.06 (0.52-8.25)	0.291
PD1+TTD vs Chemo+TTD	0.81 (0.16-4.01)	0.87 (0.41-1.87)	0.93 (0.16-5.52)	0.937
PDL1 vs Chemo+TTD	0.72 (0.22-2.34)	0.59 (0.25-1.37)	1.22 (0.28-5.24)	0.790
PDL1+CTLA4 vs Chemo+TTD	0.53 (0.14-1.83)	0.51 (0.18-1.41)	1.04 (0.20-5.39)	0.979
CTLA4+Chemo vs CTLA4	0.55 (0.10-2.89)	1.58 (0.48-5.34)	0.35 (0.04-2.76)	0.301
PD1 vs CTLA4	0.63 (0.18-2.11)	0.38 (0.19-0.76)	1.66 (0.40-6.81)	0.485
PD1+CTLA4 vs CTLA4	2.43 (0.70-9.28)	0.59 (0.28-1.23)	4.12 (0.93-18.3)	0.055
PDL1 vs CTLA4	0.31 (0.09-0.98)	0.91 (0.41-2.04)	0.34 (0.08-1.44)	0.132
PDL1+CTLA4 vs CTLA4	0.35 (0.13-1.00)	0.77 (0.26-2.18)	0.45 (0.10-1.98)	0.303
Placebo vs CTLA4	0.21 (0.08-0.52)	0.15 (0.05-0.45)	1.40 (0.33-5.93)	0.646
PD1+Chemo vs PD1	3.45 (1.78-6.85)	1.53 (0.66-3.56)	2.25 (0.77-6.63)	0.137
PD1+CTLA4 vs PD1	1.88 (1.07-3.40)	1.03 (0.45-2.31)	1.83 (0.67-4.97)	0.220
PD1+TTD vs PD1	1.82 (0.99-3.45)	1.67 (0.86-3.29)	1.09 (0.44-2.72)	0.849
PDL1+TTD vs PD1	3.94 (0.69-35.1)	0.99 (0.48-2.03)	3.98 (0.49-32.3)	0.155
Placebo vs PD1	0.36 (0.12-1.00)	0.37 (0.12-1.05)	0.97 (0.21-4.43)	0.978
TTD vs PD1	0.80 (0.31-2.13)	1.32 (0.76-2.26)	0.61 (0.20-1.83)	0.369
PD1+CTLA4 vs PD1+Chemo	0.58 (0.10-2.50)	0.60 (0.30-1.26)	0.97 (0.17-5.63)	0.962
PD1+TTD vs PD1+Chemo	0.58 (0.13-2.37)	0.68 (0.34-1.38)	0.85 (0.17-4.27)	0.859
PD1+TTD vs PD1+CTLA4	1.25 (0.50-3.09)	0.98 (0.51-1.91)	1.28 (0.41-3.93)	0.671
Placebo vs PD1+CTLA4	0.29 (0.08-0.98)	0.19 (0.06-0.51)	1.53 (0.29-7.93)	0.599
TTD vs PD1+CTLA4	0.87 (0.32-2.37)	0.67 (0.34-1.30)	1.30 (0.39-4.33)	0.667
TTD vs PD1+TTD	0.79 (0.45-1.37)	0.48 (0.23-1.00)	1.65 (0.65-4.14)	0.281
PDL1+Chemo vs PDL1	0.27 (0.01-2.99)	2.23 (1.03-4.90)	0.12 (0.01-2.32)	0.104
PDL1+TTD vs PDL1	0.52 (0.21-1.27)	2.00 (0.70-6.21)	0.26 (0.06-1.07)	0.055
Placebo vs PDL1	0.01 (0.01-0.08)	0.33 (0.14-0.76)	0.03 (0.01-0.12)	**0.014**
TTD vs PDL1	0.76 (0.26-2.16)	1.01 (0.49-2.06)	0.75 (0.21-2.70)	0.651
TTD vs PDL1+TTD	0.83 (0.40-1.66)	1.21 (0.29-5.02)	0.69 (0.14-3.38)	0.640

The ratio of risk ratios for direct and indirect estimates with their 95% confidence intervals. A confidence interval including 1 indicates there is no statistically detectable inconsistency between direct and indirect treatment estimates. Bold values denote statistical significance.

RAEs, renal adverse events; TTD, Targeted therapy drug; Chemo, Chemotherapy; PD-1, programmed cell death 1; PD-L1, programmed cell death ligand 1; CTLA4, cytotoxic T-lymphocyte antigen 4.

Table S9. Subgroup analyses and meta-regression of network meta-analysis

Treatment regimens	Primary and secondary outcomes				Meta-regression		
	**All-grade RAEs**	**Grade 3-5 RAEs**	**All-grade AKI**	**Grade 3-5 AKI**	**Median age**	**Male**	**Cancer type**
PD1+CTLA4	**1.60 (1.02-2.55)**	0.97 (0.50-1.89)	0.95 (0.49-1.91)	0.71 (0.28-1.74)	1.54 (0.97-2.46)	**1.64 (1.04-2.63)**	**1.82 (1.17-2.86)**
PD1+TTD	**1.76 (1.14-2.75)**	1.65 (0.82-3.43)	1.37 (0.73-2.66)	1.49 (0.61-3.69)	**1.77 (1.14-2.79)**	**1.79 (1.15-2.84)**	**1.91 (1.25-2.95)**
PD1+Chemo	**2.69 (1.62-4.42)**	**1.86 (1.05-3.55)**	**2.21 (1.08-4.99)**	**2.29 (1.05-5.60)**	**2.63 (1.58-4.33)**	**2.61 (1.56-4.33)**	**2.49 (1.53-4.03)**
PDL1	1.29 (0.79-2.12)	1.15 (0.62-2.14)	1.31 (0.60-3.00)	1.23 (0.48-3.12)	1.21 (0.73-2.01)	1.25 (0.76-2.07)	1.33 (0.83-2.14)
PDL1+CTLA4	1.10 (0.55-2.16)	0.81 (0.29-2.17)	1.20 (0.41-3.66)	0.62 (0.12-2.72)	1.07 (0.53-2.10)	1.06 (0.52-2.10)	1.13 (0.58-2.15)
PDL1+TTD	1.20 (0.62-2.34)	0.68 (0.29-1.47)	1.11 (0.38-3.48)	0.81 (0.23-2.57)	1.16 (0.60-2.29)	1.20 (0.61-2.38)	1.27 (0.67-2.40)
PDL1+Chemo	**2.49 (1.25-4.95)**	1.86 (0.78-4.67)	1.40 (0.54-3.91)	1.31 (0.40-4.26)	**2.53 (1.26-5.04)**	**2.41 (1.18-4.94)**	**2.51 (1.30-4.85)**
CTLA4	**1.95 (1.10-3.51)**	**3.04 (1.30-7.20)**	2.20 (0.58-8.06)	2.46 (0.57-10.6)	**1.90 (1.06-3.42)**	**1.94 (1.08-3.65)**	**1.97 (1.11-3.51)**
CTLA4+Chemo	2.12 (0.85-5.37)	2.11 (0.79-5.78)	2.66 (0.47-16.7)	2.98 (0.52-20.4)	2.10 (0.84-5.36)	2.05 (0.82-5.22)	2.07 (0.86-5.06)
TTD	1.17 (0.73-1.87)	0.55 (0.28-1.05)	**0.46 (0.21-0.93)**	**0.23 (0.08-0.56)**	1.12 (0.69-1.82)	1.18 (0.73-1.92)	1.31 (0.82-2.06)
Chemo	1.34 (0.95-1.91)	1.45 (0.89-2.49)	1.40 (0.78-2.82)	1.59 (0.80-3.47)	1.39 (0.97-1.98)	1.27 (0.88-1.83)	1.37 (0.98-1.93)
Chemo+TTD	**2.07 (1.16-3.77)**	1.45 (0.63-3.34)	1.69 (0.67-4.34)	1.53 (0.56-4.44)	**2.01 (1.11-3.67)**	**1.97 (1.08-3.65)**	**1.93 (1.09-3.46)**
Placebo	**0.37 (0.17-0.76)**	**0.32 (0.10-0.83)**	**0.20 (0.03-0.99)**	**0.10 (0.01-0.70)**	**0.36 (0.17-0.74)**	**0.37 (0.17-0.76)**	**0.40 (0.19-0.79)**
PD1	Reference	Reference	Reference	Reference	Reference	Reference	Reference
DIC	354	333	235	226	345	346	353
Number of studies	95	90	63	62	93	92	95
Heterogeneity[a]	0.20 (0.09-0.40)	0.12 (0.00-0.57)	0.12 (0.00-0.64)	0.17 (0.00-1.00)	0.21 (0.10-0.41)	0.21 (0.10-0.42)	0.17 (0.07-0.36)

Data are risk ratios with their 95% confidence intervals after adjusting covariates: median age, proportion of male and cancer type.

[a]Heterogeneity was assessed using the posterior median between trial variance, tau^2.

Significant results are in bold.

RAEs, renal adverse events; AKI, acute kidney injury; TTD, Targeted therapy drug; Chemo, Chemotherapy; PD-1, programmed cell death 1; PD-L1, programmed cell death ligand 1; CTLA4, cytotoxic T-lymphocyte antigen 4; DIC, deviance information criterion.

Table S10. SUCRA values stratified by cancer types

Treatment regimens	Respiratory system		Digestive system		Urogenital system		Hematologic system		Skin	
	Ranking	SUCRA	Ranking	SUCRA	Ranking	SUCRA	Ranking	SUCRA	Ranking	SUCRA
**PD1**	8	45.8	6	44.6	6	34.9	6	39.6	1	83.4
**PD1+CTLA4**	12	33.3	-	-	4	55.8	10	17.4	-	-
**PD1+TTD**	11	35.1	8	16.0	7	32.1	7	36.7	5	44.2
**PD1+Chemo**	10	36.6	7	34.2	-	-	2	92.9	6	29.8
**PDL1**	4	74.3	2	73.6	8	15.6	-	-	4	55.9
**PDL1+CTLA4**	5	61.5	3	73.0	-	-	-	-	2	78.9
**PDL1+TTD**	1	79.3	9	14.0	3	57.1	11	14.0	-	-
**PDL1+Chemo**	13	32.5	-	-	2	73.9	-	-	-	-
**CTLA4**	9	41.1	5	49.2	-	-	8	32.9	7	0.4
**CTLA4+Chemo**	14	11.0	-	-	-	-	5	48.4	-	-
**Placebo**	2	78.7	1	94.5	-	-	3	73.9	-	-
**TTD**	3	77.6	-	15.2	5	51.1	8	32.9	-	-
**Chemo**	6	60.7	4	59.9	1	76.9	1	96.9	-	-
**Chemo+TTD**	7	48.6	-	-	-	-	4	65.6	3	57.2

SUCRA, surface under the cumulative ranking; TTD, Targeted therapy drug; Chemo, Chemotherapy; PD-1, programmed cell death 1; PD-L1, programmed cell death ligand 1; CTLA4, cytotoxic T-lymphocyte antigen 4.

Table S11. Sensitivity analyses of network meta-analysis

Treatment regimens	All-grade RAAEs	Exclude 5 single-center studies	Exclude 19 studies with sample size less than 100	Exclude 16 studies using CTCAE version 3.0	Exclude 11 studies with data of phrase 1 trials	Exclude 12 studies performed before 2013	Exclude 19 studies with high overall risk of bias	Exclude 11 studies with fibrosis, PRF and CKD patients
PD1+CTLA4	**1.60 (1.02-2.55)**	**1.58 (1.00-2.55)**	1.61 (0.99-2.68)	**1.67 (1.04-2.74)**	1.81 (0.92-3.60)	1.66 (0.92-3.04)	**1.72 (1.03-2.89)**	**1.73 (1.10-2.78)**
PD1+TTD	**1.76 (1.14-2.75)**	**1.65 (1.00-2.77)**	1.70 (0.88-3.31)	**1.86 (1.17-3.02)**	**1.67 (1.01-2.83)**	**1.73 (1.02-2.99)**	1.68 (0.85-3.35)	**1.75 (1.17-2.68)**
PD1+Chemo	**2.69 (1.62-4.42)**	**2.65 (1.58-4.39)**	**2.70 (1.57-4.57)**	**2.58 (1.50-4.39)**	**2.64 (1.45-4.73)**	**2.60 (1.42-4.65)**	**2.67 (1.55-4.50)**	**2.88 (1.81-4.54)**
PDL1	1.29 (0.79-2.12)	1.27 (0.77-2.11)	1.12 (0.66-1.92)	0.93 (0.50-1.73)	1.38 (0.82-2.35)	1.30 (0.76-2.20)	0.92 (0.51-1.67)	1.20 (0.68-2.11)
PDL1+CTLA4	1.10 (0.55-2.16)	1.08 (0.54-2.15)	1.16 (0.55-2.39)	0.87 (0.38-1.94)	1.08 (0.48-2.31)	1.07 (0.51-2.19)	1.03 (0.49-2.15)	0.80 (0.33-1.86)
PDL1+TTD	1.20 (0.62-2.34)	1.17 (0.59-2.33)	1.08 (0.51-2.28)	2.08 (0.86-5.53)	1.29 (0.63-2.68)	1.24 (0.60-2.62)	2.09 (0.85-5.65)	1.11 (0.54-2.36)
PDL1+Chemo	**2.49 (1.25-4.95)**	**2.46 (1.22-4.93)**	**2.45 (1.18-5.03)**	**2.08 (1.00-4.34)**	**2.55 (1.24-5.24)**	**2.46 (1.17-5.08)**	**2.32 (1.13-4.77)**	**4.01 (1.75-9.36)**
CTLA4	**1.95 (1.10-3.51)**	**1.92 (1.07-3.50)**	**1.91 (1.01-3.63)**	2.09 (0.96-4.60)	1.64 (0.84-3.24)	1.84 (0.93-3.67)	1.76 (0.89-3.48)	**1.86 (1.06-3.33)**
CTLA4+Chemo	2.12 (0.85-5.37)	2.11 (0.84-5.34)	2.11 (0.82-5.52)	1.13 (0.18-7.50)	2.85 (0.94-8.81)	5.19 (0.30-204)	2.70 (0.90-8.37)	2.24 (0.95-5.45)
TTD	1.17 (0.73-1.87)	1.12 (0.68-1.87)	1.16 (0.66-2.05)	1.30 (0.76-2.25)	1.19 (0.71-2.00)	1.23 (0.68-2.23)	1.31 (0.71-2.45)	1.18 (0.76-1.84)
Chemo	1.34 (0.95-1.91)	1.33 (0.94-1.91)	1.34 (0.93-1.94)	1.12 (0.75-1.68)	1.38 (0.95-2.00)	1.34 (0.92-1.96)	1.27 (0.87-1.85)	1.43 (1.00-2.05)
Chemo+TTD	**2.07 (1.16-3.77)**	**2.04 (1.13-3.55)**	**2.15 (1.08-4.37)**	**2.00 (1.01-4.01)**	**2.04 (1.11-3.81)**	**2.06 (1.11-3.89)**	**2.05 (1.09-3.97)**	**1.95 (1.13-3.44)**
Placebo	**0.37 (0.17-0.76)**	**0.37 (0.17-2.33)**	**0.36 (0.16-0.76)**	**0.30 (0.11-0.76)**	**0.35 (0.15-0.74)**	**0.39 (0.16-0.87)**	**0.35 (0.15-0.73)**	**0.38 (0.18-0.74)**
PD1	Reference	Reference	Reference	Reference	Reference	Reference	Reference	Reference
DIC	354	339	296	293	315	314	282	312
Number of studies	95	90	76	79	84	83	76	84
Heterogeneity[a]	0.20 (0.09-0.40)	0.21 (0.09-0.43)	0.24 (0.10-0.48)	0.24 (0.10-0.48)	0.24 (0.10-0.48)	0.25 (0.10-0.51)	0.24 (0.10-0.47)	0.13 (0.03-0.34)

[a] Heterogeneity was assessed using the posterior median between trial variance, tau2.

Significant results are in bold.

RAEs, renal adverse events; CTCAE, Common Terminology Criteria for Adverse Events; PRF, postrenal failure; CKD, chronic kidney disease; TTD, Targeted therapy drug; Chemo, Chemotherapy; PD-1, programmed cell death 1; PD-L1, programmed cell death ligand 1; CTLA4, cytotoxic T-lymphocyte antigen 4; DIC, deviance information criterion.
